# Antibody to gp41 MPER Alters Functional Properties of HIV-1 Env without Complete Neutralization

**DOI:** 10.1371/journal.ppat.1004271

**Published:** 2014-07-24

**Authors:** Arthur S. Kim, Daniel P. Leaman, Michael B. Zwick

**Affiliations:** Department of Immunology and Microbial Science, The Scripps Research Institute, La Jolla, California, United States of America; University of Zurich, Switzerland

## Abstract

Human antibody 10E8 targets the conserved membrane proximal external region (MPER) of envelope glycoprotein (Env) subunit gp41 and neutralizes HIV-1 with exceptional potency. Remarkably, HIV-1 containing mutations that reportedly knockout 10E8 binding to linear MPER peptides are partially neutralized by 10E8, producing a local plateau in the dose response curve. Here, we found that virus partially neutralized by 10E8 becomes significantly less neutralization sensitive to various MPER antibodies and to soluble CD4 while becoming significantly more sensitive to antibodies and fusion inhibitors against the heptad repeats of gp41. Thus, 10E8 modulates sensitivity of Env to ligands both pre- and post-receptor engagement without complete neutralization. Partial neutralization by 10E8 was influenced at least in part by perturbing Env glycosylation. With unliganded Env, 10E8 bound with lower apparent affinity and lower subunit occupancy to MPER mutant compared to wild type trimers. However, 10E8 decreased functional stability of wild type Env while it had an opposite, stabilizing effect on MPER mutant Envs. Clade C isolates with natural MPER polymorphisms also showed partial neutralization by 10E8 with altered sensitivity to various gp41-targeted ligands. Our findings suggest a novel mechanism of virus neutralization by demonstrating how antibody binding to the base of a trimeric spike cross talks with adjacent subunits to modulate Env structure and function. The ability of an antibody to stabilize, destabilize, partially neutralize as well as alter neutralization sensitivity of a virion spike pre- and post-receptor engagement may have implications for immunotherapy and vaccine design.

## Author Summary

As vaccination, immunoprophylaxis and immunotherapies are becoming increasingly feasible approaches to combat HIV/AIDS, understanding the activity of relevant anti-HIV antibodies is crucial. Antibody 10E8 defines a key vulnerability on the envelope spikes of a vast majority of HIV isolates but mechanisms of resistance to this neutralizing antibody are incompletely understood. Our findings show how partial neutralization of HIV can occur through apparent partial occupancy by 10E8 of HIV spikes that is accompanied by specific, antibody mediated effects on spike stability, infectivity and sensitivity to various inhibitors of HIV. We reveal a previously unappreciated mechanism of spike-antibody recognition where consequences on viral infectivity by 10E8 binding are dependent on interactions between subunits of the virion spike that modulate its stability and recognition properties. HIV vaccine development and immunoprophylaxis involving 10E8-like antibodies and their target, the gp41 MPER, may have to consider functional relationships involving the MPER and antibody occupancy at the base of trimeric spikes.

## Introduction

Advances in both vaccine development and immunoprophylaxis are needed to combat HIV/AIDS [1]–[3]. Both of these strategies target the viral envelope glycoprotein spike (Env), which is a trimer of gp120-gp41 heterodimers. HIV-1 Env is functionally labile [4], [5], heterogeneously glycosylated [6]–[9] and phylogenetically diverse [www.hiv.lanl.gov]. The membrane proximal external region (MPER) of HIV-1 is an important target on the transmembrane subunit gp41 as it is linked to a highly conserved sequence motif and epitopes of several broadly neutralizing antibodies [10]–[12]. However, a general inability to elicit broadly neutralizing antisera to these and other conserved epitopes on HIV-1 Env by vaccination has led to deeper investigation of the relevant Env-antibody interactions [1]–[3], [13].

Models of the MPER typically focus on peptide monomers, either on micelles, lipid bilayers or in solution [14]–[17]. Broadly neutralizing MPER antibodies, 2F5, 4E10, Z13e1, and the extremely potent 10E8 antibody have helped characterize the native MPER. Crystal structures of these antibodies in complex with MPER monomers have revealed distinct local conformations while detailed structural information of the MPER on HIV-1 Env trimers is currently lacking [10], [18]–[22]. Hydrophobic CDR H3s seem to be crucial for MPER antibody neutralization [18], [23]–[25]. In sequential binding models, the hydrophobic H3s of 2F5 and 4E10 first engage the viral membrane leading to binding of a membrane-embedded MPER monomer [15], [25]. A somewhat different model shows the H3 of MPER antibodies dipping between the membrane and a six-helix bundle form of gp41 [26], while a precise role for membrane in neutralization by 2F5 has been challenged [27]. Remarkably, 10E8 neutralizes HIV-1 with ≥10-fold greater potency than previously described MPER antibodies [10]. Although 10E8 seems to show weak binding to membranes the relationship between this activity and neutralization is incompletely understood [28], [29].

Although antibodies can reach an occupancy level of three per Env spike [30], [31], studies have suggested that a single antibody is sufficient for HIV-1 neutralization [32], [33]. Limits to occupancy are also possible, as antibody PG9 binds to just one gp120 protomer of the spike in an asymmetric manner [34]. MPER antibodies are the most potent of the described neutralizing antibodies to gp41, and can bind to unliganded Env of sensitive isolates, but not typical neutralization-resistant isolates [35]–[39]. Engagement of host CD4 by Env stabilizes a site on gp120 for coreceptor (i.e. CCR5 or CXCR4) and also reveals elements of gp41, including the MPER, N-heptad repeat (NHR) and C-heptad repeat (CHR) regions [40]–[42]. Antibody stoichiometry following receptor engagement is poorly understood, but a short kinetic time window, steric blocks and flexibility in gp41 together appear to affect the potency of 2F5, 4E10, Z13e1 and certain fusion inhibitors post-receptor engagement [19], [38], [42]–[44].

Models have depicted the MPER at the base of unliganded spikes where it might interact with other elements of gp41 [45]. Indeed, mutations to the MPER can destabilize Env spikes [4], and both 2F5 and 4E10 can cause spikes to shed gp120 [39]. The extreme potency of neutralization by 10E8 is not adequately explained by its affinity for MPER peptide, which is comparable to that of less potent neutralizers, 2F5, 4E10 and Z13e1 [10]. Moreover, whereas some MPER mutations diminish antibody binding to peptide and abrogate neutralization (*e.g.* W672A with 4E10), others diminish peptide binding but enhance neutralization (*e.g.* I675A with Z13e1) [46], [47]. These and other findings have led to the conclusion that neutralizing MPER epitopes involve elements besides current crystallographic defined linear epitopes [11], [12], [48].

We wished to gain new insight into MPER mediated neutralization using 10E8. We discovered an unexpected mechanism in which HIV-1 becomes partially neutralized by 10E8 wherein potency is high against a ‘neutralizable’ fraction of virus infectivity. Whereas 10E8 readily occupies all three protomers of wild type unliganded spikes it partially and inefficiently occupies MPER mutant unliganded spikes, indicative of a hindrance to further occupancy by 10E8 on adjacent subunit(s). Here, 10E8 seems to bind but remarkably not fully inhibit HIV-1 spikes from mediating infection of target cells. Moreover, with the ‘non-neutralizable’ fraction of virus infectivity we find that 10E8 can kinetically alter stability and ligand-binding properties of Env spikes pre-receptor engagement as well as post-receptor engagement in distinct ways. These features define a novel mechanism of HIV-1 neutralization involving the MPER, accessibility by antibody to the MPER, and interactions between MPER and adjacent elements of Env. The novel mechanism described for 10E8 conceivably might also influence HIV-1 facing 10E8-like antibodies *in vivo*, and so has relevance to immunotherapy and vaccine approaches.

## Results

### HIV-1 MPER mutants are only partially resistant to 10E8 neutralization

**Figure 1:**
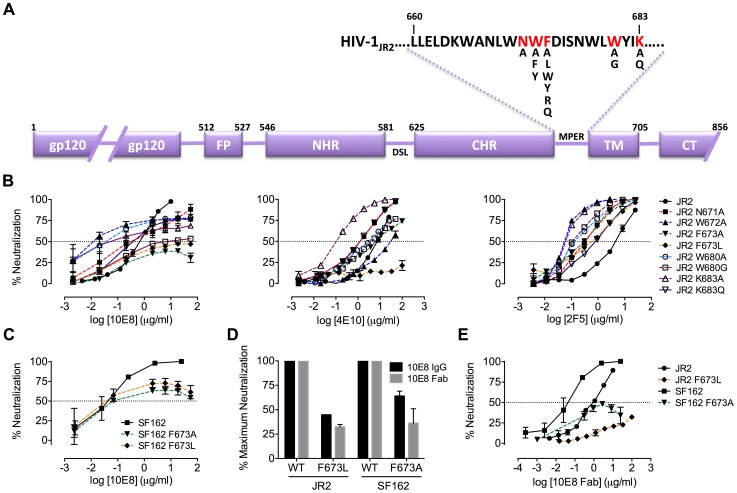
Partial neutralization of HIV-1 JR2, SF162 and corresponding MPER mutants by 10E8. (**A**) Diagram of the MPER and surrounding domains of HIV-1 gp41 with residues found to be important for neutralization by 10E8 highlighted in red. (**B**) Neutralization of JR2 and cognate MPER mutants against broadly neutralizing antibody 10E8 (left) performed side-by-side with 4E10 (middle) and 2F5 (right). (**C**) Neutralization of SF162 wild type and F673 mutants by 10E8. (**D**) Maximum neutralization (plateau) percentages and (**E**) dose response neutralization curves of 10E8 (IgG and Fab) against JR2 and SF162 wild type and F673 Ala mutants. Error bars are from two independent experiments [n = 2] performed in duplicate using TZM-bl target cells.

MPER mutants of JR2 were shown previously to be incompletely neutralized at high concentrations of 10E8 [10]. A molecular basis for partial neutralization by 10E8 has not been described so we decided to investigate. These mutants, and some newly engineered mutants with naturally occurring MPER polymorphisms [49], [50], were tested in neutralization assays against 10E8, 4E10 and 2F5 (**Table S1**). Of 21 Ala mutants covering positions 660–680 of the MPER, only mutants W672A, F673A, W680A and K683A were partially neutralized by 10E8, *i.e.* neutralization curves plateaued with less than full neutralization (**Figure 1A** **;** data not shown). One mutant, N671A, was less sensitive to 10E8 but became fully neutralized by 10E8 at high concentration (**Figure 1B**). Mutants containing natural polymorphisms F673L, W680G and K683Q also showed partial neutralization by 10E8 with plateaus at 30–80% maximum neutralization and relatively shallow curves (**Figure 1B**). We note that at very high concentrations of 10E8 (>50 µg/ml) the plateau of some curves occasionally inflected and showed a downward slope; however this effect was not reproducible between experiments and plateaus were also observed with no downward slope (see **Figure S1** and below). Importantly, in all cases partial neutralization was consistently reproducible. The nature of amino acid substitution also had an effect on 10E8 neutralization. Mutants containing conservative substitutions of aromatics for aromatics (F673W/Y and W672Y/F) were fully neutralized whereas substitutions from hydrophobics to hydrophilics (F673R/Q) were partially neutralized with maximum plateaus at 50–60% (**Figure S2**). Antibody 2F5 to an MPER epitope upstream of 10E8 neutralized all of the mutants completely and more potently than wild type virus (**Figure 1B**), as previously reported [47]. With 4E10 we were unable to determine whether full neutralization was achievable at high concentration due to limiting antibody reagent. Nevertheless, 4E10 neutralized mutant F673A with a shallow slope indicating that as antibody concentration increases neutralization becomes less efficient (**Figure 1B**). The natural polymorphism F673L notably conferred almost complete resistance to 4E10 at both IC_90_ and IC_50_. Remarkably, no single mutation tested imparted complete resistance to 10E8.

Having verified partial neutralization by 10E8 of JR2, a Tier 2-like primary isolate, we decided to test a more sensitive strain, SF162, for which direct access by MPER antibodies to unliganded Env has been reported [35]. We put particular focus on the naturally occurring mutation, F673L, as it has been observed in multiple HIV-1-infected individuals [49], [50], and shows clear partial neutralization by 10E8 in the JR2 background. Notably, F673L is observed in 0.97% of 4009 reported sequences of Envs among primary isolates [http://www.hiv.lanl.gov/content/sequence/QUICK_ALIGN/QuickAlign.html]. In most models of the MPER, F673 is found buried, either in the paratopes of 10E8 and 4E10 [10], [51], in peptide-embedded micelles [14], in lipid bilayers [15] or within a MPER peptide homotrimer [52]. F673A also nearly knocks-out 10E8 binding to MPER peptide [10]. Hence, we envisioned that the mechanism behind partial neutralization of MPER mutants by 10E8 could be most readily elucidated using substitutions of F673. 10E8 partially neutralized mutants F673L and F673A in the hypersensitive SF162 Env background, which indicated that partial neutralization by 10E8 was not JR2 specific and can also occur with a Tier 1 strain (**Figure 1C**).

### MPER mutation does not generally alter gp41 fusion kinetics

Partial neutralization using dose-saturating concentrations of 10E8 conceivably might be due to an MPER mutant having enhanced fusion kinetics that limits the time in which 10E8 can act post-receptor engagement. We therefore tested the sensitivity of mutant F673L to fusion inhibitors C34 and 5-Helix, which act post-receptor engagement on NHR and CHR regions of the gp41 pre-fusion intermediate, respectively. We found that IC_50_s of JR2 F673L against C34 and 5-Helix (IC_50_ = 0.9 µg/ml and IC_50_ = 4.1 µg/ml, respectively) were very similar to that of wild type HIV-1 for these inhibitors (IC_50_ = 1.2 µg/ml and IC_50_ = 6.1 µg/ml, respectively); this was also true in the SF162 Env background (data not shown). In fact, the F673L mutants were hypersensitive to MPER antibodies 2F5 and Z13e1 (**Figure 1B** **and** **2**). Hence, fusion kinetics are unlikely to be accelerated by the gp41 mutation F673L, although exposure of the MPER may be increased. We also found no correlation between infectivity of virus stocks and partial neutralization, as well as no obvious correlation between reported affinities for MPER mutant peptides and neutralization of cognate mutant viruses by 10E8 [10] (data not shown). Moreover, 10E8 partially neutralized mutant F673L using target cells bearing FcγRI receptors (**Figure S3A and D**). FcγRI improves on-rates of antibodies against receptor-activated gp41, particularly MPER antibodies [53]. The above results suggest that partial neutralization by 10E8 is not a result of the pre-fusion intermediate of gp41 having generally altered fusion kinetics.

**Figure 2:**
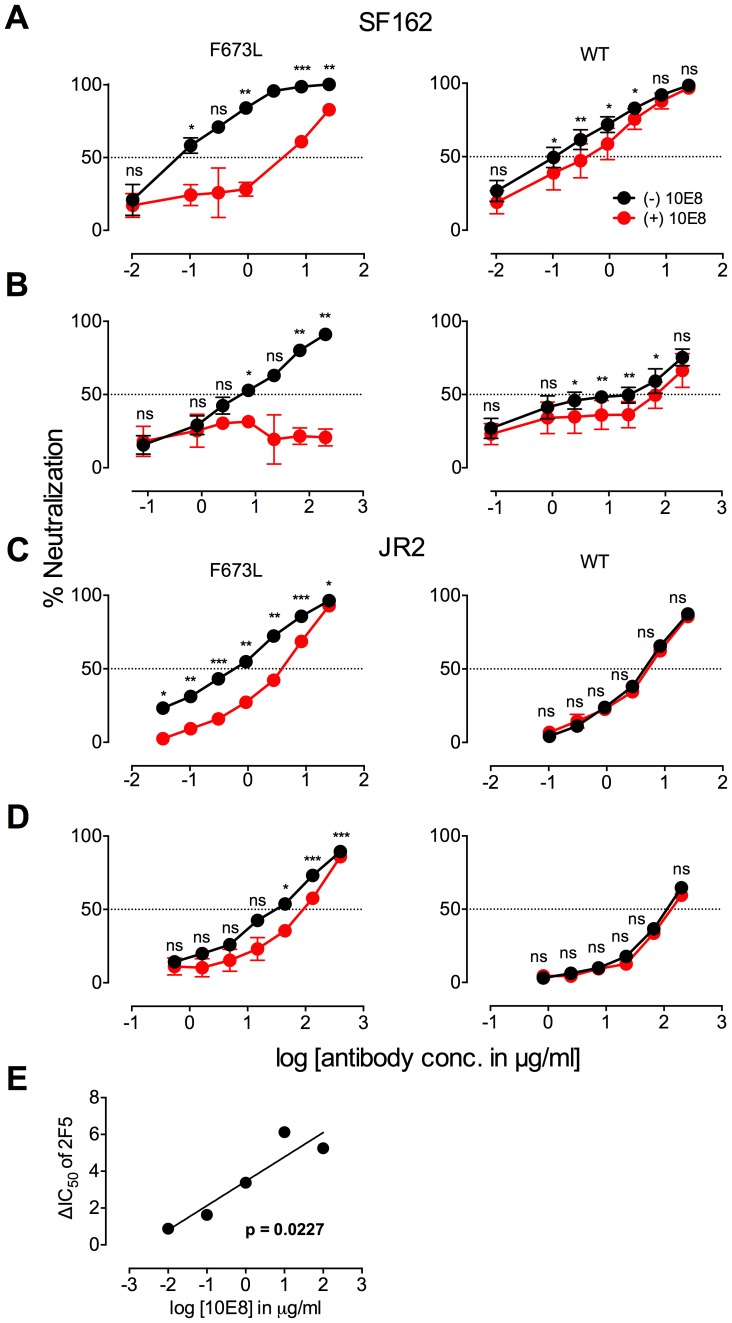
Presence of 10E8 diminishes neutralization sensitivity of HIV-1 to 2F5 and Z13e1. The maximum infectivity of virus in presence or absence of 10E8 was normalized to 100% for mutant F673L (left panels) and wild type HIV-1 (right panels); 10E8 was held constant at 10 µg/ml and the IC_50_ for F673L mutants and wild type viruses, respectively. The 10E8 resistant fraction of viral infectivity was assayed against (**A** and **C**) 2F5 and (**B** and **D**) Z13e1. Neutralization curves in black and red indicate absence and presence of 10E8, respectively, for (**A** and **B**) SF162 and (**C** and **D**) JR-FL. Experiments were performed in duplicate in at least two independent experiments, except for wild type SF162 that was tested in four independent experiments (n = 4). Statistical significance was determined for each pair of points along the dose response curves (in presence or absence of 10E8) using an unpaired t test and p-values were corrected using the Sidak-Bonferroni method for multiple comparisons. Symbols above each pair of data points represent the p-values (*, p<0.05; **, p<0.01; ***, p<0.001; ns, not significant). (**E**) Statistical significance of the change in IC_50_ of 2F5 against JR2 F673L due to the presence of 10E8 was determined using linear regression analysis.

### 10E8 partial neutralization is neither target cell-type nor antibody format dependent

We considered that characteristics specific to TZM-bl target cells, or heterogeneity or molecular size of 10E8 IgG might be causing partial neutralization with 10E8. However, incomplete neutralization of HIV-1 mutant F673L was also observed using U87.CD4.CCR5 cells and HOS.CD4.CCR5 cells with plateaus similar to that of TZM-bls (**Figure S3B, C, and D**). We also used 10E8 IgG produced both transiently in 293 cells and using a stable CHO-K1 cell line as well as Fab 10E8 prepared by enzymatic digestion. Both in-house 10E8 IgG preparations, a sample from the NIH ARRRP and the Fab 10E8 molecule all showed partial neutralization against the F673L mutant, however the potency with the Fab was found to be lower (**Figure 1D and E**; data not shown). 10E8 IgG can aggregate at concentrations above ∼0.7 mg/ml [29]. However, the neutralization plateaus are observed at 10E8 concentrations 100-fold lower than this aggregation point; moreover, we found that insoluble aggregates of 10E8 also produced partial neutralization curves that were similar to that of soluble 10E8 (**Figure S1B**). Hence, partial neutralization by 10E8 appears to occur independently of target cell type, antibody format or solubility state of the antibody

### N-linked glycosylation alters neutralization of MPER mutant by 10E8

**Figure 3:**
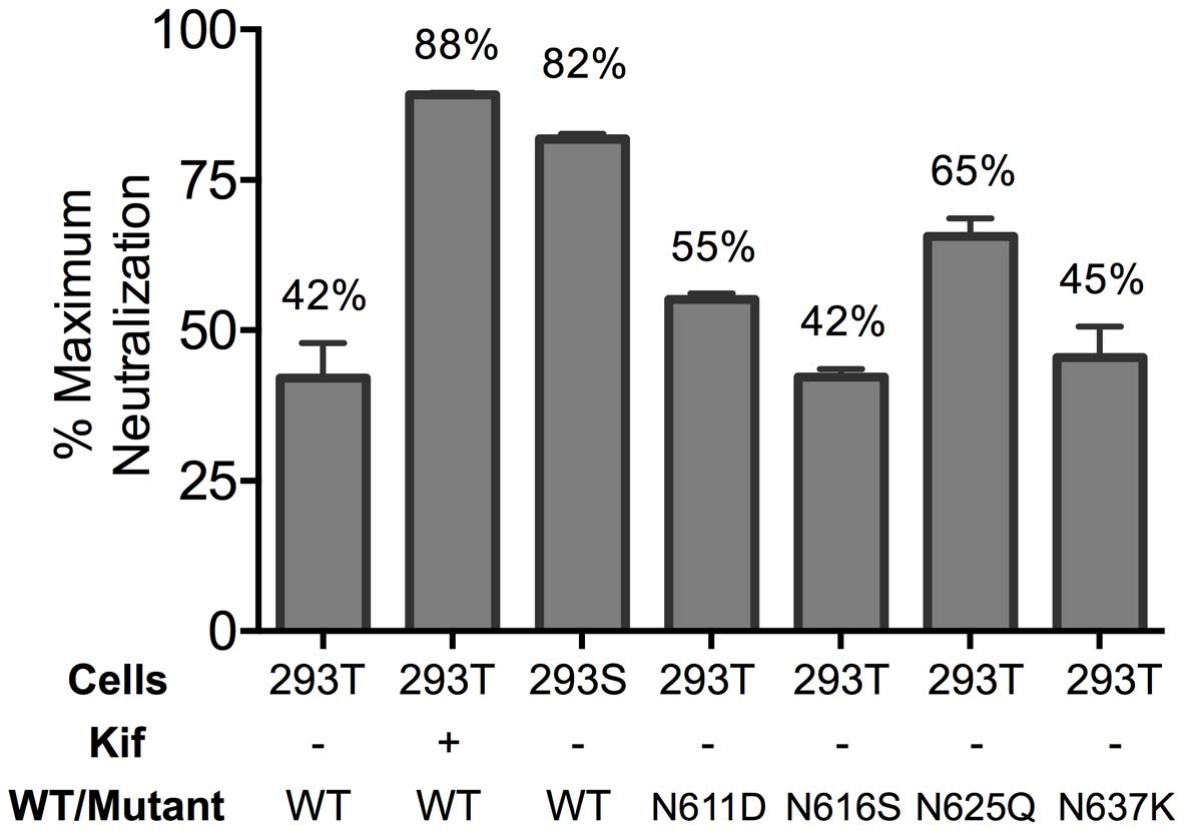
Glycosylation state of MPER mutant HIV-1 Env influences extent of maximum neutralization by 10E8. The JR2 F673L mutant was subjected to various glycosylation conditions and the corresponding viruses were tested in a neutralization assay against 10E8 using TZM-bl target cells to determine the maximum neutralization percentage.

Partial neutralization could indicate differences in glycosylation that result in sensitive and resistant viral subpopulations. The crystallographic defined epitope of 10E8 has only protein elements, but glycans conceivably could affect MPER accessibility. We therefore produced virions in GnTI^-/-^ (293S) cells and in 293T cells treated with kifunensine (Kif), which results in relatively homogeneous Man_5_-Man_9_ and Man_9_ glycan residues, respectively [6], [54]. MPER mutant viruses produced in GnTI^-/-^ or Kif-treated cells were tested in a neutralization assay and were still partially neutralized by 10E8, but plateaus were shifted from 42% (293T, no Kif) to 82% and 88% neutralization, respectively (**Figure 3** **; Figure S4A and C**). Kif treatment also slightly reduced viral infectivity and cleavage efficiency (by ∼3-fold, and from >95% to 80–85%, respectively), while it caused gp120 and gp41 to run slightly faster on SDS-PAGE (**Figure S4** and data not shown). However, these effects of Kif on processing and function of Env were equal against wild-type and mutant so the changes in maximum neutralization by 10E8 are not a simple function of diminished infectivity or cleavage. To see if glycans on gp41 were responsible for limiting neutralization by 10E8, we individually ablated the four N-linked glycosylation sequons (NGS) in gp41 on an F673L mutant background and tested these double mutants in a neutralization assay. None of the NGS mutations affected the sensitivity of the F673L mutant to control antibodies 2F5 or 4E10; however, N625Q increased the maximum level of 10E8 neutralization from 41% to 65% (**Figure 3** **; Figure S4B**). The other three NGS knockouts were no more or less sensitive to 10E8 neutralization. Whereas contributions from other glycans or factors besides glycosylation cannot be ruled out, the results above suggest that complex glycan on Env and the glycan at N625 of gp41, can significantly affect the maximum neutralization achieved by 10E8.

### Presence of 10E8 alters recognition properties across multiple sites of HIV Env

**Figure 4:**
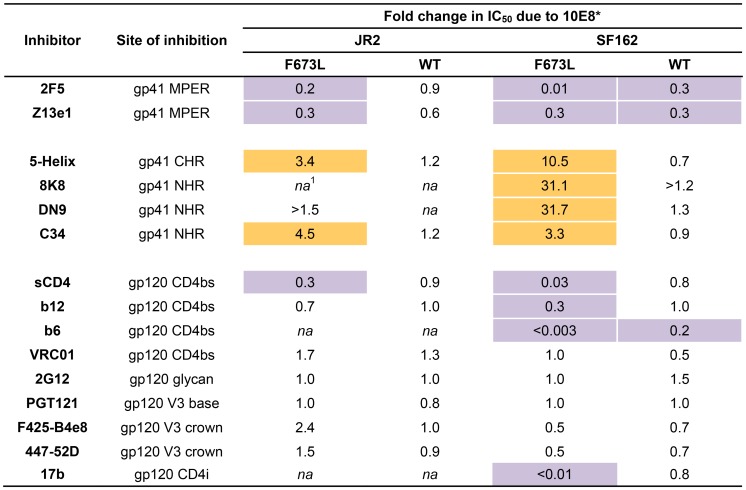
Effect of presence of 10E8 on the sensitivity of HIV-1 and corresponding F673L mutant to various ligands (IC_50_). Asterisk (*) indicates fold change in IC_50_ was calculated using the equation: (IC_50_ without 10E8/IC_50_ with 10E8). Purple highlight shows when neutralization potency is decreased by greater than three fold and gold highlight shows when neutralization potency is increased greater than three fold. Hashtag (#) indicates that the data is not applicable (*na*) as an IC_50_ was not reached at concentrations tested.

To further investigate partial neutralization by 10E8, we considered whether 10E8 might somehow occupy MPER mutant Env trimers of the neutralization resistant fraction of virus without fully blocking their ability to mediate infection. We speculated that the presence of 10E8 might also affect neutralization at epitopes beside that of 10E8. Focusing on the F673L mutant in both JR2 and SF162 Env backgrounds, we chose a fixed saturating concentration of 10E8 IgG that was within the maximum plateau of neutralization, and varied that of several fusion inhibitors (*e.g.* 5-Helix and C34) as well as antibodies to gp41 (*e.g.* 2F5, Z13e1, 8K8 and DN9) or gp120 (*e.g.* sCD4, b12, b6, VRC01, 2G12, PGT121, F425-B4e8, 447-52D, and 17b). For comparison, we similarly fixed a somewhat lower sub-neutralizing concentration of 10E8 IgG to use against wild type JR2 and wild type SF162 viruses. Indeed, the results confirmed our speculation. Remarkable differences were observed in the potency of a number of different inhibitors and antibodies against Env due to the presence of 10E8, seen most significantly against the F673L mutants (**Figure 4**), but notably also to a lesser extent against wild type SF162, but not at all with wild type JR2 (**Figure 2** **,** **4** **,** **5** **and** **6**). To our knowledge, this is the first example in which an antibody binds to an infectious viral spike and specifically alters its neutralization sensitivity involving a variety of different epitopes and sites of fusion inhibition.

**Figure 5:**
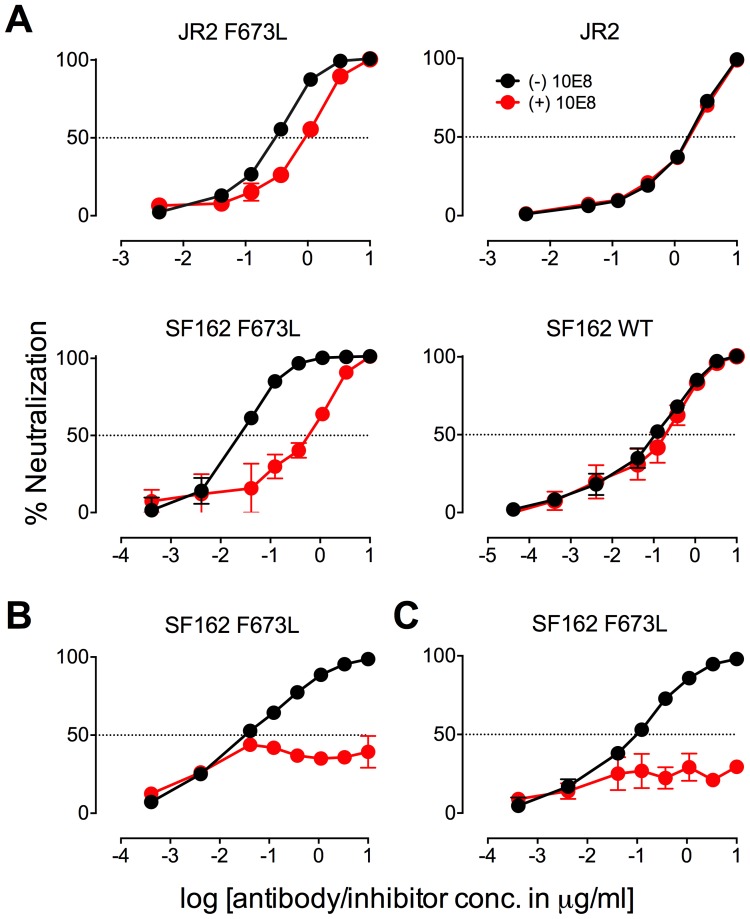
Presence of 10E8 alters sensitivity of HIV-1 to soluble CD4 as well as to antibodies against CD4 and coreceptor binding sites. The presence (red) or absence (black) of 10E8 at 10 µg/ml or the IC_50_ for F673L mutants and wild type viruses, respectively, affects the neutralization sensitivity of cognate virus to (**A**) soluble CD4, (**B**) b6 IgG and (**C**) 17b IgG.

**Figure 6:**
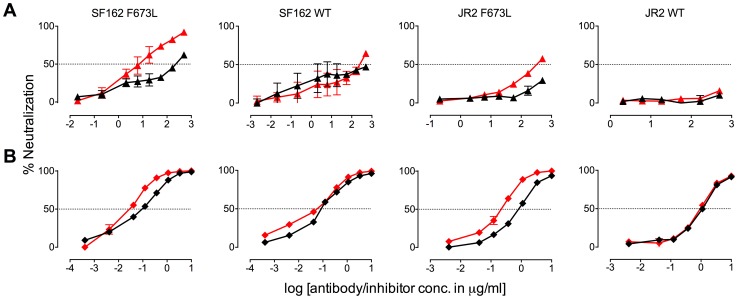
Presence of 10E8 alters sensitivity of HIV-1 to post-attachment fusion inhibitors. The presence (red) or absence (black) of 10E8 at 10 µg/ml or the IC_50_ for F673L mutants and wild type viruses, respectively, affects the neutralization sensitivity of cognate virus to (**A**) DN9 IgG and (**B**) C34 peptide.

There are several notable observations to make on the neutralization experiments performed in the presence and absence of 10E8. First, the effect of 10E8 on the IC_50_ of certain ligands can be very significant, *i.e.* over an order of magnitude with 2F5 (**Figure 2** **and** **4**), which cannot be explained from the 40–50% change in relative viral infectivity after treatment with 10E8 alone (**Figure 1**)**.** Second, the presence of 10E8 decreases sensitivity to MPER antibodies, as might be expected with overlapping epitopes (**Figure 2**). Third, the presence of 10E8 hyper-sensitizes the virus to fusion inhibitors C34 and 5-Helix, as well as antibodies 8K8 and DN9, all of which target sites on the heptad repeats of a receptor-activated, pre-bundle form of gp41 that do not overlap with that of 10E8 (**Figure 4** **and** **5**). Fourth, still other antibodies like 2G12 and PGT121 show no change in potency in the presence of 10E8 (**Figure 4**). Fifth, 10E8 decreases the apparent potency of soluble CD4 (sCD4), as well as CD4 binding site antibody b6 and coreceptor site antibody 17b against the otherwise sensitive MPER mutant of SF162, suggesting that 10E8 restricts conformational changes required for binding by sCD4, b6 and 17b (**Figure 4** **and** **5** **;** see below). This effect was highly pronounced for weakly neutralizing antibodies 17b and b6 (*i.e.* >10-300-fold decreases in neutralization of SF162 F673L) that might be particularly sensitive to increases in Env rigidity. Finally, the presence of 10E8 can even affect the IC_50_ of antibodies against wild type Tier 1A isolate SF162 in the absence of the F673L mutation, and the directionality of the shift in IC_50_s with wild type SF162 caused by 10E8 is the same as with the F673L mutant for cognate antibody, though the magnitude in the shift is less (**Figure 2** **and** **4**). To determine if other inhibitors altered MPER recognition, we tested neutralization of mutant F673L by MPER and CD4bs antibodies in the presence of sub-neutralizing concentrations of 4E10, b12 or sCD4. No significant change was observed under these conditions either in the potency of these antibodies or in the magnitude of partial neutralization by 10E8 (**Table S2;** data not shown). Importantly, the presence of human serum (*e.g.* 20% or equivalent to ∼3 mg/ml IgG) did not affect the level of partial neutralization of the F673L mutant by 10E8 suggesting that the effects we observed may also occur *in vivo* (data not shown).

The results above show that the presence of 10E8 *(i)* neither makes Env globally neutralization sensitive nor globally resistant but has more specific effects on both gp41 and gp120, as well as *(ii)* modulates both receptor-naïve and receptor-activated spikes since 10E8 antagonizes ligands like sCD4 but potentiates ligands like C34, which exclusively target unliganded and receptor-activated spikes, respectively. Moreover, 10E8 modifies neutralization sensitivity of wild type HIV-1 (*e.g.* SF162). Notably implicit with the strongest of the observed effects of 10E8 on neutralization profiles of HIV-1 is the perhaps contraintuitive notion that 10E8 may be binding to most, if not all, Env spikes with at least partial subunit occupancy and without fully inhibiting their function.

### Stoichiometry of 10E8 binding to HIV-1 trimeric spikes (pre-attachment)

**Figure 7:**
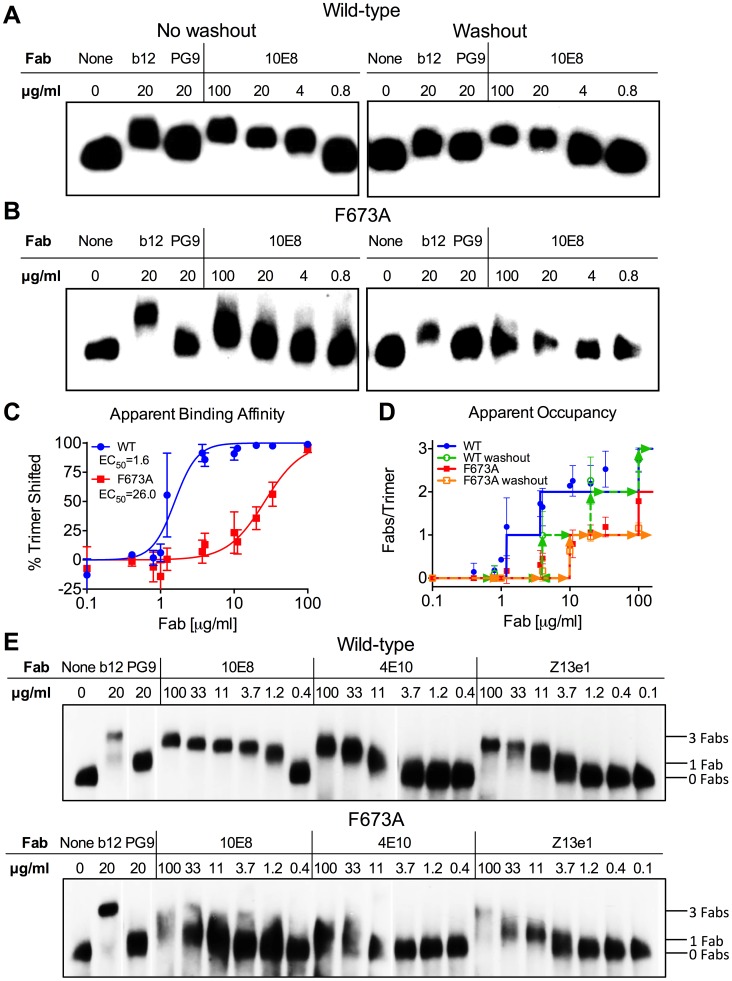
10E8 binds less efficiently to Env spikes of MPER mutant F673A compared to wild type. Fab 10E8 was incubated with JR-FL (**A**) wild type or (**B**) F673A virions. In washout experiments, virion-Fab mixtures were pelleted by centrifugation and supernatants replaced with buffer devoid of Fab. Gel mobility of Env trimers was determined using BN-PAGE and Western blot probed with a cocktail of gp120 and gp41 antibodies. (**C**) BN-PAGE gel mobility shift data were used to generate binding curves for 10E8 against detergent-solubilized Env spikes. The intensity of Env trimer band that remained unshifted at each concentration of 10E8 relative to that of an antibody-free control was determined using densitometry for eight independent blots and averaged results were plotted. (**D**) Occupancy of 10E8 binding to Env trimers at different concentrations was quantified by measuring the distance between midpoints of Fab-shifted vs untreated bands, and plotting the result as a function of the distance shifted by Fabs b12 and PG9 that are assumed to bind three and one Fab(s) per trimer, respectively. Eight independent blots were analyzed for the “no washout” condition and three independent blots for the “washout” treatment. The averaged data points do not fall on whole numbers so a line is shown at the nearest round number of Fab occupancy to facilitate interpretation. (**E**) 10E8, 4E10 or Z13e1 Fabs were incubated with JR-FL wild type or F673A virions and resulting changes to gel mobility of Env trimers was determined using BN-PAGE and Western blot. A cocktail of gp120 and gp41 antibodies were used to probe the blot. Electrophoresis was performed using 3–8% Tris-Acetate NuPAGE gels (Invitrogen) under native conditions. Stoichiometry of binding was determined by measuring the gel mobility shift compared to Fab controls b12 and PG9.

We speculated that partial neutralization of MPER mutants by 10E8 might relate to stoichiometry of 10E8 binding to Env trimers. Addressing antibody occupancy post-receptor engagement is not straightforward. However, blue native (BN) PAGE can be useful for addressing stoichiometry with unliganded Env [33]. We used BN-PAGE to separate wild type and F673A Env in complex with 10E8 and probed Western blots using Env-specific antibodies [9], [55]. Fab 10E8 was used for these experiments since it partially neutralizes but cannot crosslink spikes which can confound measurements. JR-FL E168K was used for Env because it forms homogeneous trimers, and can be probed using the trimer specific antibody PG9 [9], [56]. Fab 10E8 caused a quantitative shift of the entire visible band corresponding to JR-FL trimers for both wild-type and F673 mutant virions (**Figure 7**). We found no evidence of 10E8-unreactive Env trimers. Wild type JR-FL spikes were shifted farther on the gel and also at lower concentrations of 10E8 than mutant F673A spikes (**Figure 7**). Similar results were seen with F673 mutants in the SF162 background (data not shown). Fab Z13e1 readily shifted F673A trimers, consistent with its ability to efficiently neutralize this mutant, while stoichiometry of 4E10 binding to F673A appeared to be reduced similar to 10E8, consistent with 4E10's ability to achieve an IC_50_ but not an IC_80_ against F673A mutant HIV-1 [47] (**Figure S5**). Compared to control Fabs PG9 and b12, which bind to one and three gp120 subunit(s) on Env [30], [34], respectively, 10E8 shifted wild type Env spikes to the same degree as b12. However, 10E8 shifted the F673A trimer band by more than PG9 and by less than b12. These data would suggest that two subunits of MPER mutant Env would be occupied by 10E8 at a concentration of ∼10 µg/ml that is near its local maximum in neutralization (**Figure 1A**). The same concentration by contrast would fully neutralize and saturate all three subunits of wild type virus. It should be cautioned however that BN-PAGE analyses measure binding to unliganded trimers while 10E8 neutralizes in large part post-receptor engagement. Notwithstanding, the above results suggest that 10E8 binds to wild type trimers with both higher stoichiometry and higher apparent affinity than F673 mutant spikes.

Because 10E8 can bind to Env before or after detergent-solubilization, or both, we performed a washout step prior to adding detergent so that only Fab already bound to native trimers would remain. This procedure had a modest effect on 10E8 binding to wild type JR-FL spikes, verifying that 10E8 can bind to unliganded Env on virions, although it likely binds more efficiently post-solubilization (**Figure 7**). However, 10E8 occupancy of F673A Env was clearly reduced from two to one Fab per spike by the washout step (equivalent shift to PG9). This result implies that 10E8 binds inefficiently to mutant spikes in the membrane, although the lower affinity interaction may also allow Fab 10E8 molecules to fall off during solubilization and the PAGE procedure. We note that the washout step also reduced Fab b12 occupancy suggesting that binding affinity may be limited for at least one of the b12 molecules on the trimer. Nevertheless, it appears from both neutralization assays and BN-PAGE analyses that binding of 10E8 molecules to unliganded MPER mutant Env is blocked from reaching full subunit occupancy, either directly or indirectly, but that no such limitation to 10E8 occupancy exists with wild type Env.

### 10E8 functionally stabilizes MPER mutant Env

**Figure 8:**
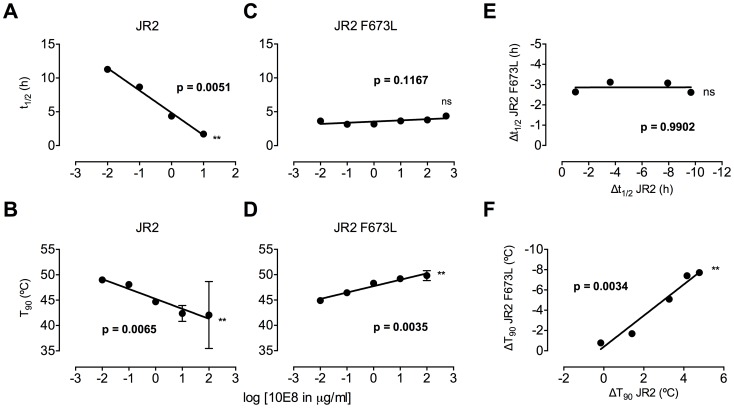
Enhancement by 10E8 of the functional stability of MPER mutant F673L and corresponding diminution of functional stability of wild type HIV-1 Env. (**A**) Half-life (t_1/2_) of infectivity decay at 37°C and (**B**) thermostability of JR2 wild type and (**C and D, respectively**) F673L mutant in the presence of incremental concentrations of 10E8. Thermostability was determined using a heat gradient to obtain the temperature at which viral infectivity drops by 90% (T_90_). (**E**) Relationship of 10E8-induced changes in infectivity decay (Δt_1/2_) of JR2 F673L mutant is plotted against that of wild type JR2. (**F**) The 10E8-induced change in thermostability (ΔT_90_) of wild type JR2 is inversely correlated with that of the F673L mutant.

Ruprecht et al previously showed that 2F5 and 4E10 cause Env spikes to shed gp120 subunits, gradually inactivating HIV-1 in an irreversible process that takes several hours [39]. We anticipated that 10E8 would also alter Env stability, particularly as we found it alters the neutralization sensitivity profile of partially neutralized virus. First, we incubated wild type HIV-1 (JR2) at physiological temperature in the presence or absence of 10E8 or 2F5 over a time course and then measured infectivity. A sub-saturating concentration of 10E8 (*i.e.* 0.1 µg/ml) decreased the half-life of wild type JR2 from 13.6 h to 8.7 h, indicating that it too destabilizes functional Env spikes over time (**Figure 8A**). Antibody 2F5 decreased the half-life of JR2 as anticipated, from 13.6 h to 8.9 h (data not shown). JR2 stability was also evaluated using a thermostability assay that determines the temperature (T_90_) at which an Env variant of HIV-1 loses 90% of its infectivity in one hour [4]. In line with the physiological decay results, 10E8 strongly reduced the T_90_ of wild type JR2 from 49°C to 43°C in a dose dependent manner, indicating that 10E8 decreases wild type Env stability at both physiologic and elevated temperatures (**Figure 8B**).

**Figure 9:**
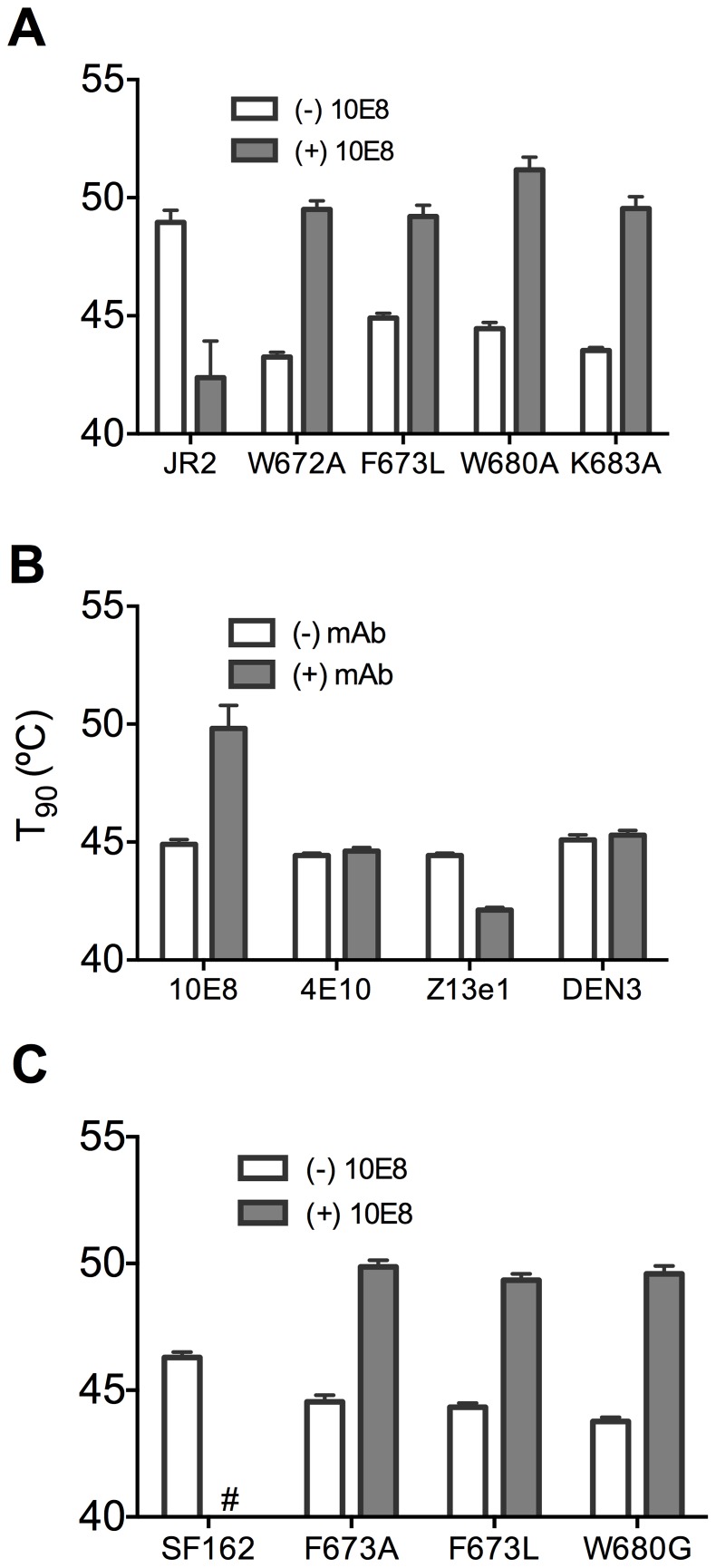
Presence of 10E8 enhances functional thermostability of multiple MPER mutants of JR2 and SF162 within the 10E8 linear epitope. (**A**) The presence of 10E8 at a concentration in the maximum neutralization plateau (10 µg/ml) enhances functional thermostability (T_90_) of multiple MPER mutants of JR2. Hash tag (#) indicates insufficient infectivity. (**B**) The effect on the functional thermostability of JR2 F673L by the presence of control antibodies 4E10, Z13e1 and DEN3, which are non-neutralizing, neutralizing and irrelevant antibodies against this mutant, respectively. 4E10 and Z13e1 were used at 50 µg/ml and DEN3 was used at 100 µg/ml. (**C**) Functional thermostability of SF162 in the presence of 10E8 at a concentration in the maximum neutralization plateau (10 µg/ml).

In contrast to its effect on wild type JR2, 10E8 altered the functional stability of F673L with a different pattern. Thus, at a low concentration range of 10E8 (∼0.01–1.0 µg/ml) infectivity decay of F673L at physiologic temperature remained relatively constant but at high concentrations (∼500 µg/ml) 10E8 reproducibly increased its half-life slightly from 3.6 h to 4.4 h (**Figure 8C**). This was in contrast to 2F5 that decreased the already short half-life of the mutant virus from 7.1 h to 5.8 h (data not shown). Surprisingly, in the thermostability assay, 10E8 significantly increased the thermostability (T_90_) of F673L from 45°C to 50°C in a dose dependent manner (**Figure 8D**). Hence, the change in T_90_ caused by 10E8 with mutant F673L shows a strongly significant inverse correlation with that of wild type JR2 (p = 0.0034; **Figure 8F**). To see whether stabilization by 10E8 of the MPER mutant was restricted only to F673L, we assayed other MPER mutants that were partially neutralized by 10E8. The presence of 10E8 strongly increased the stability (T_90_) of mutants W672A, W680A and K683A by 5–6°C (**Figure 9A**). Controls 4E10 (50 µg/ml) and DEN3 (100 µg/ml) had no effect on thermostability of JR2 F673L whereas the presence of Z13e1 (50 µg/ml) decreased the thermostability of F673L (**Figure 9B**), the latter result being consistent with Z13e1's ability to fully neutralize this mutant. The presence of 10E8 also increased the thermostability of several MPER mutants in an SF162 background (**Figure 9C**). Hence, while 10E8 decreases the thermostability of wild type JR2 it increases the thermostability of Envs disrupted by mutations at different positions along the MPER. Stabilization of functional Env by a neutralizing antibody is to our knowledge unprecedented.

### Physical stability of 10E8-Env complexes

**Figure 10:**
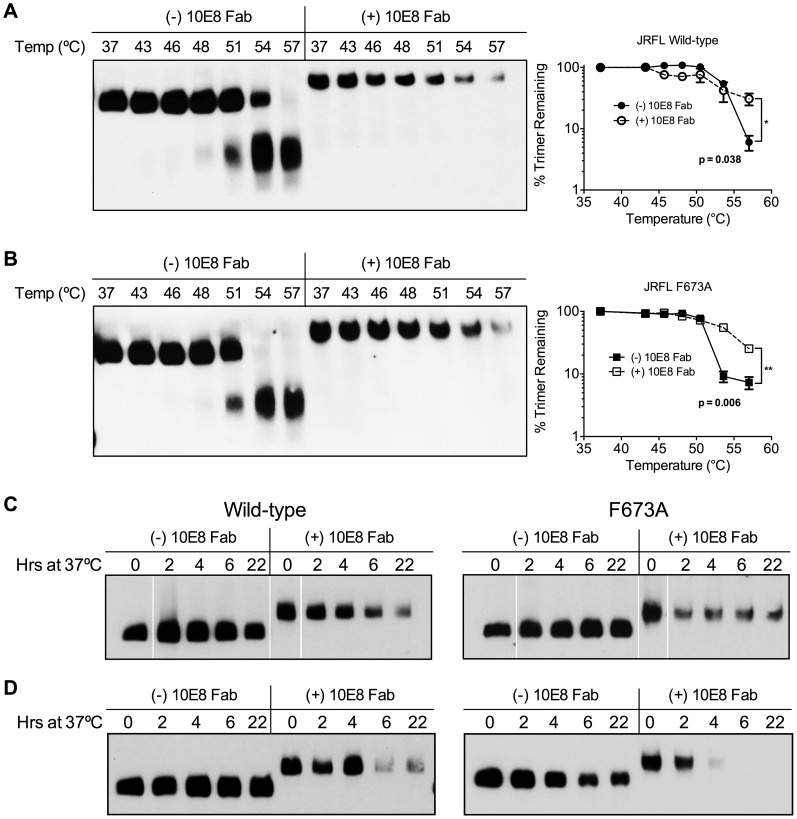
10E8 enhances physical stability of Env trimers against heat-induced denaturation. JR-FL (**A**) wild type and (**B**) F673A mutant virions were exposed to increasing temperatures in the absence (leftmost lanes) or presence of 100 µg/ml 10E8 Fab (rightmost lanes). Following detergent solubilization, the oligomeric state of Env was determined using BN-PAGE and Western blot probed with anti-gp41 antibodies. The intensity of the Env trimer bands from each lane were quantified and plotted as the percentage of trimer remaining relative to that incubated at 37°C (far right). (**C**) JR-FL wild type or F673A virions or (**D**) detergent-solubilized Env was incubated in the presence or absence of 10E8 Fab over a time course at 37°C, and the oligomeric state of Env was analyzed using BN-PAGE Western blot.

We next assessed whether effects of 10E8 on Env function directly relate to effects on the oligomeric state of Env. First, wild type JR-FL and F673A mutant virions were subjected to a heat gradient in the presence or absence of 10E8 Fab and Env was analyzed using BN-PAGE. Unexpectedly, we found that binding of 10E8 stabilized a fraction of both wild type and MPER mutant Env trimers against temperatures that caused dissociation of Env spikes in the absence of 10E8 (**Figure 10A and B**). However, in the presence of 10E8 the band corresponding to the Env trimer did grow fainter after exposure to higher temperatures. Fading of the Env trimer band after 57°C treatment was observed to a similar degree when the Western blot was stained using different combinations of antibodies against multiple epitopes on gp41 or gp120 (**Figure S7**; data not shown). Hence, when 10E8-Env complexes bound by 10E8 are exposed to elevated temperatures they are stabilized, but may become altered to be less prominent on BN-PAGE analysis, perhaps through aggregation, while unbound spikes dissociate into individual subunits.

To explore stability effects further, virions were also incubated at physiological temperature and then analyzed using BN-PAGE. Here, for up to 22 hours there was no detectable decay of JR2 spikes or MPER mutant spikes, as seen previously [4]. However, when 10E8 was present most of the trimer band faded at 37°C over time with some trimer remaining; decay products were again not visible (**Figure 10C**). Similar fading on addition of 10E8 was observed when virion-associated Env was incubated at 37°C in detergent (DDM), except that the trimer band disappeared more rapidly (**Figure 10D**). Thus, 10E8 alters physical stability of functional Env trimers over time at physiological temperature, perhaps causing them to aggregate, but a fraction of 10E8-bound Env remains relatively stable on membrane. We conclude that at physiological temperature 10E8 kinetically stabilizes F673 mutant Env into one population that is active and one that is inactive, but into inactive conformations only for wild type spikes. We also conclude that effects of 10E8 on Env trimer stability depend on level of subunit occupancy as well as temperature and time of incubation.

### 10E8 neutralizes MPER mutant Env function primarily post-attachment

**Figure 11:**
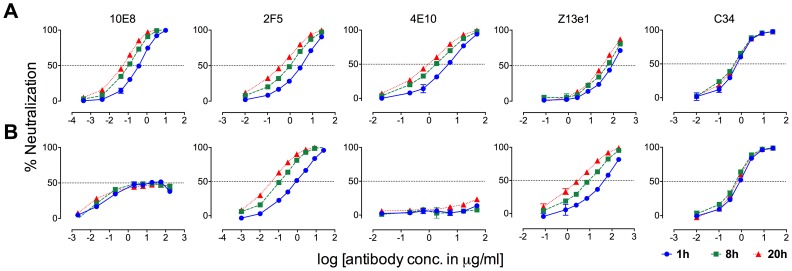
Kinetics of neutralization of JR2 F673L mutant by 10E8 and C34. (**A**) Wild type JR2 and (**B**) mutant F673L were incubated with MPER antibodies or fusion inhibitor C34 to the NHR of gp41 over a time course at 37°C before adding onto target (TZM-bl) cells.

MPER antibodies neutralize HIV-1 in large part by binding to Env post-receptor engagement [37], [42], [57]–[59], whereas neutralization pre-attachment varies between HIV-1 isolates [35]. Partial neutralization of F673 mutants by 10E8 must be limited both pre and post attachment to host cells as it occurs in saturating amounts of 10E8 maintained throughout the assay. However, the relative limits to 10E8 neutralization prior to and following receptor engagement might be different. In time course experiments, we found that 10E8 neutralization increases (IC_50_ decreases) roughly 10-fold with SF162 and JR2 wild type HIV-1 when virus is pre-incubated with 10E8 up to 20 hours rather than the standard 1 hour prior to adding to target cells (**Figure 11A**, left panel; data not shown). This result is consistent with Env destabilizing properties (pre-attachment) previously reported for 2F5 and 4E10 (**Figure 11A**, middle panels) [39], and as demonstrated above for 10E8 (**Figure 8A and B**). However, a different effect was observed with F673L mutant Env. In this case, rather than 10E8 neutralization becoming more potent over time, both potency and the maximum percentage of F673 mutant virus neutralized were maintained during the 20-hour pre-incubation (**Figure 11B**, left panel). The potency of fusion inhibitors 5-Helix (data not shown) and C34, which only act post attachment [41], [42], remained unchanged throughout the assay as expected (**Figure 11**, rightmost panel). We also found that viral stock age had no effect on the level of partial neutralization (**Figure S7**), which is consistent with our findings above that mobility on BN-PAGE of 10E8-bound F673A spikes did not change with incubation times of up to 22 hours prior to running the gel (**Figure 10C**). We conclude that whereas 10E8 readily occupies and gradually inactivates wild type Env, it inefficiently occupies and stabilizes infectivity of MPER mutant Env over time.

10E8 neutralization of JR-FL is reportedly relatively resistant to pre-attachment washout [10], and our experiments using JR2 concurred with this (**Figure S8**). However, neutralization of F673L virus by 10E8 could be completely washed away prior to adding virions to target cells (**Figure S8**). This agrees with our BN-PAGE data that showed that the apparent affinity of 10E8 on the MPER mutant spike is relatively low and is readily washed off (**Figure 7**). Because 10E8 partially neutralizes JR2 F673 mutant in standard assay format we conclude that neutralization of the mutant primarily occurs post-attachment.

### 10E8 alters functional properties of natural clade C Envs without complete neutralization

**Figure 12:**
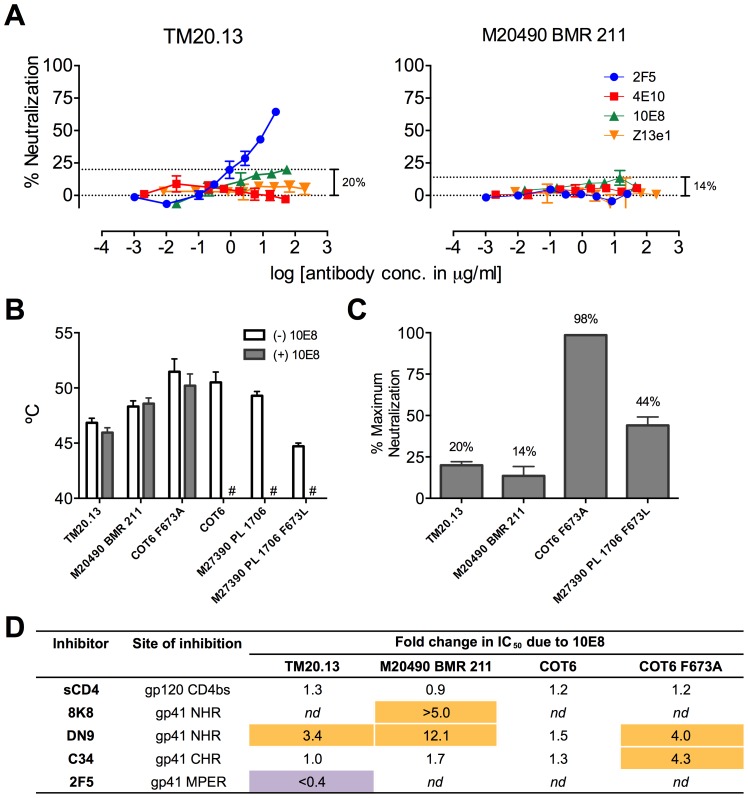
Functional effects of 10E8 on HIV-1 clade C variants including those with a naturally occurring L673 residue. (**A**) Partial neutralization by 10E8 of clade C isolates TM20.13 and M20490 BMR 211 using the TZM-bl assay. (**B**) Effect of the presence of 10E8 at a saturating concentration (10 µg/ml) on thermostability (T_90_) of clade C isolates or cognate F673 mutants. Hash tag (#) indicates insufficient infectivity due to lability or neutralization. (**C**) Percentage of maximum neutralization by 10E8 of various clade C isolates or cognate F673 mutants. (**D**) Effect of the presence of 10E8 on neutralization sensitivity of clade C isolates or cognate F673 mutants to various ligands. Fold change in IC_50_ was calculated using the equation: (IC_50_ without 10E8/IC_50_ with 10E8). Purple highlight shows when neutralization potency is decreased by greater than three-fold and gold highlight shows when neutralization potency is increased greater than three-fold. *nd*, not determined.

MPER polymorphisms such as L673 occur naturally in different individuals infected with clade C isolates (**Table S1**) [49], [50]. L673 mutations have been used herein with well-characterized clade B envelopes, JR2 and SF162, in which MPER mutations including F673L are destabilizing (**Figure 8**) [4]. We speculated that isolates that naturally incorporate a Leu at position 673 might have co-evolved to compensate for instability in the MPER and might therefore respond differently to 10E8. We first tested two clade C isolates that contain L673, TM20.13 [50] and M20490 BMR 211 [49], which have previously been shown to be resistant to 4E10 and Z13e1 (**Table S1;** **Figure 12A**). Antibody 10E8 showed rather weak neutralizing activity against these isolates. However, at high concentrations of 10E8 (∼10–50 µg/ml) partial neutralization was observed plateauing at 14% and 20% for the two isolates; the effect was specific to 10E8 as no such effect was seen using 4E10 (**Figure 12A**) [49], [50]. In addition, consistent with our speculation that these Envs may have adapted to the presence of L673 and therefore might respond differently to 10E8, 10E8 had little to no effect on the thermostability (T_90_) of TM20.13 or M20490 BMR 211 Env spikes (**Figure 12B**). However, the presence of 10E8 did show specific effects on sensitivity to neutralizing ligands with these two isolates (**Figure 12D**). Neutralization of TM20.13 by 2F5 was hindered in the presence of 10E8 (2F5 does not neutralize M20490 BMR 211), whereas neutralization by both 8K8 and/or DN9 was enhanced when 10E8 was present. Hence, as with the clade B MPER mutants, the pre-fusion intermediate of gp41 with these clade C isolates is being stabilized by 10E8 in a conformation favorable to the gp41 inhibitors but without being fully inactivated, while access to the MPER by further antibodies is blocked. Furthermore, the presence of 10E8 did not alter sensitivity of the clade C isolates to the pre-attachment inhibitor, sCD4, which may relate to 10E8's lack of effect on the thermostability (T_90_) of the clade C Envs, as both sCD4 binding and thermostability are properties of Env in its unliganded (pre-attachment) state.

To further determine whether 10E8 would alter stability or ligand recognition of clade C viruses, we tested F673 mutants of two other clade C Envs. Thus, an F673A mutant of an otherwise 4E10 sensitive clade C isolate, COT6 [50], showed evidence of partial neutralization by 10E8 with a shallow slope and noticeable plateau in the high 98% range that was absent with its wild type Env counterpart (**Figure 12C**). Furthermore, an F673L mutant was generated for the 4E10-sensitive clade C isolate, M27390 PL 1706 [49], which was partially neutralized by 10E8 with a plateau at 44%, closer to what was observed with the clade B MPER mutants (**Figure 12C**). Further characterization of the pseudotyped Envs was more problematic as they are considerably more heterogeneous in BN-PAGE relative compared to the homogeneous JR-FL trimers [36] while Env M27390 PL 1706 also showed low infectivity. Sensitivity of the COT6 F673A mutant to ligands targeting the gp41 pre-fusion intermediate was also specifically enhanced by the presence of 10E8, whereas thermostability was not affected (**Figure 12B and D**). Thus, effects of 10E8 on pre-receptor engaged Env (*i.e.* effects of 10E8 on thermostability and sensitivity to sCD4) can vary and be distinct from effects of 10E8 on receptor-activated Env (*i.e.* effects of 10E8 on sensitivity to fusion inhibitors). Taken in sum, our analysis shows that 10E8 can alter ligand recognition properties of functional clade C Env spikes with both naturally occurring and introduced MPER mutations.

## Discussion

HIV-1 Env has evolved to sequester its most conserved surfaces from recognition by neutralizing antibodies of the host. With the MPER this likely involves steric limitations imposed by Env and viral membrane both pre- and post-engagement. However, molecular details are lacking on how MPER antibodies overcome these limitations. Here, we reveal novel mechanisms of 10E8 activity not accounted for by prior models. First, we confirmed that antibody 10E8 causes unusual partial neutralization with certain Envs. We showed that 10E8 occupied an apparent maximum of two gp41 subunits of an MPER mutant trimer instead of three subunits observed with its wild type counterpart that was fully neutralized. Clade C isolates with natural MPER polymorphisms were also partially neutralized by 10E8 suggesting that this phenotype could evolve during natural infection under antibody pressure. Second, we found that 10E8 functionally destabilizes unliganded Envs while functionally stabilizing mutant Env counterparts, the latter activity of which is unprecedented for a virus-neutralizing antibody. Third, we found that the presence of 10E8 can significantly alter the sensitivity of Env to neutralization by antibodies and inhibitors to gp120 and gp41. A quaternary model incorporating behavior of each MPER on trimeric Env we think provides for a superior account of these observed effects of MPER recognition by 10E8-like antibodies.

There has been uncertainty as to whether MPER antibodies act on a pre-hairpin intermediate [57] or on a late six-helix bundle form of gp41 [26]. In our studies, the presence of 10E8 enhanced sensitivity of HIV-1 to fusion inhibitors C34 and 5-Helix, which must act prior to six-helix bundle formation. We conclude from these results that, at a minimum, 10E8 acts on a pre-hairpin intermediate of gp41. While it also remains possible that 10E8 can fall off during conformational changes caused by receptor engagement, dose-saturating concentrations of 10E8 were maintained throughout the entry process making this possibility less likely.

In the unliganded state, 10E8 can functionally stabilize mutant Envs to heat and physiological decay, and can also inhibit neutralization by sCD4 and CD4bs antibodies. However, these pre-attachment effects were limited to certain unstable Envs. In contrast, the clade C isolates in which L673 occurred naturally were not stabilized to heat in the presence of 10E8 and only ligands that bind post-attachment had activities affected by 10E8 binding. MPER antibodies bind to unliganded Env better with variants that adopt a more open conformation, but with many primary isolates can only bind post-attachment [35]. Although mutation F673L in JR2 and SF162 backgrounds did not make the Envs globally hypersensitive to neutralization to every ligand, F673L did amplify effects of 10E8 both pre- and post-attachment. Meanwhile, clade C Envs that were less reliant on the MPER for stability only appeared to be accessible to 10E8 following CD4 engagement. Whereas MPER mutations can be disrupting [4], [47], [60], compensatory mutations could have developed in these clade C Envs that uphold fitness and stability of Env in the presence of 10E8. Considering the lack of sequence homology between the isolates and that individual mutations often destabilize Env, a molecular basis for the differences in observed effects of 10E8 on different Envs will be difficult to isolate [4], [9], [61]. Perhaps longitudinal studies that follow Env mutations in face of 10E8-like antibody selection pressure in different individuals might provide insight.

Since Ala mutations to residues W672, F673, W680 and K683 in JR2 all caused similar partial neutralization as well as altered sensitivity to heat and ligands in presence of 10E8, it seems that a more general disruption of the MPER is sufficient for these effects. These mutations would affect recognition of the CDR H3 and adjacent residues of 10E8 based on existing structural data [10]. Thus, 10E8 binding to wild type Env may stabilize the MPER in a conformation that is incompatible with membrane fusion. The above mutations would decrease affinity of 10E8 for unliganded mutant Env as our washout experiments indicated, and as they also diminish 10E8 binding to MPER peptides (*e.g.* 10^2^-10^6^-fold drop in IC_50_) [10]. Weak 10E8 binding may fail to fully inactivate Env, and instead may stabilize at least a portion of the Env population into conformations capable of mediating viral entry. Interestingly, conservative mutations to the MPER (**Figure S2**) such as W672F and F673W destabilize Env JR2 but do not lead to partial neutralization presumably because high affinity of 10E8 for the MPER is maintained [62].

One antibody is typically sufficient to neutralize one HIV-1 spike [32], [34]. However, 10E8 significantly altered functional properties of Envs at concentrations in which our BN-PAGE analyses showed all observable trimeric Env was bound by 10E8. These results contra-intuitively suggest that 10E8 can occupy Env without abrogating its function. Heterogeneity in the Env population could provide explanations for how this might occur. However, experiments that perturbed glycosylation of Env showed that glycan heterogeneity can contribute to but not fully account for partial neutralization. Another explanation relates to the asymmetric nature of Env occupied by one or two 10E8 antibodies. We speculate that whether an Env spike is blocked or not by 10E8 may depend on the spatial relationship between the gp41 subunit(s) bound by 10E8 and the gp120 subunit(s) that engage host cell receptors. The MPER acts at a late step during fusion (e.g. expansion of the fusion pore [60]) in which gp41 subunits participate in a monomer-trimer equilibrium [63]. Thus, MPERs on adjacent subunits may serve partially redundant functions [64], so that occupancy by antibody under certain conditions might only retard and not fully block fusion. Better tools and atomic-level structural information on relevant conformational states of Env are needed before firmer conclusions can be drawn.

How 10E8 can diminish neutralization by other MPER antibodies cannot be completely clear without detailed structural information. However, recent structures of disulfide-stabilized soluble gp140 trimers omit the MPER [21], [22], and the structure of the MPER following receptor activation is also unknown. But the structures do show a considerable distance between points where MPERs join the trimer (∼30 Å). We therefore prefer an allosteric, or “trimer constraining” model, to explain neutralization interference between MPER antibodies and 10E8 mediated alteration of ligand sensitivity more generally (**Figure S9**). This would also explain why saturation of the mutant spike by 10E8 does not fully block 2F5 neutralization and why the effect also occurs with ligands that bind distal to 10E8. Flexibility of the MPER [19] would presumably allow propagation of conformational changes to other regions of Env upon antibody binding; functional links between the MPER, NHR and DSL regions have also been described [65], [66]. MPER mutations might also lead to an exchange of the MPER between adjacent subunits and membrane. On binding to the spike, 10E8 may stabilize conformations in which unoccupied subunits have diminished affinity for certain ligands and increased affinity for others. The propagation of conformational changes from bound to unbound protomers might explain why binding of 10E8 to one gp41 protomer reduces apparent affinity of additional MPER antibodies to other protomers as opposed to a model in which 10E8 itself is the steric block to further antibodies.

Our results are most consistent with a model in which antibody and MPER interact and function in the specific context of trimeric Env. First, there is a known lack of correlation between neutralization and antibody binding to monomeric MPER peptides [10], [17], [47], [67]. Our own attempts to correlate IC_50_s or maximum neutralization percentages of 10E8 to 10E8-peptide affinity data by Huang et al produced no obvious relationships (unpublished observations). Second, the epitopes of 2F5 and 4E10 are not well exposed on resting primary spikes suggesting an unmasking of elements of Env upon CD4 engagement that allows antibody recognition [35]–[37], [57]. Third, the MPER is enriched with hydrophobic residues that are typically found in the hydrophobic interior of proteins. Fourth, even conservative mutations to hydrophobic residues in the MPER destabilize some Envs as if they engaged in specific protein-protein rather than protein-lipid interactions [4]. Fifth, MPER disrupting mutations can enhance sensitivity of HIV-1 to MPER antibodies [4], [46], [47], [65]; conversely, selective tightening of subunit interactions diminishes neutralization by MPER antibodies [9]. Sixth, quaternary interactions between hydrophobic elements at the base of the spike could help explain why a hydrophobic tip on CDR H3 seems to be required for neutralization by MPER antibodies as its insertion would be energetically favorable and likely disruptive [10], [23]–[27], [39]. Seventh, that MPER antibodies promote gp120 shedding suggests an opposite force on the MPER keeping gp120 on the unliganded spike. Eighth, sCD4 enhances MPER exposure, which shows reciprocal links between gp120 and the MPER.

Examination of the literature turned up no equivalent mechanism to 10E8 partial neutralization. Although several different neutralization mechanisms have been described for MPER antibodies, including antibody-induced shedding of gp120 [39], pre-attachment and post-attachment antibody binding [35], [37], [42], [58], [59], [68], these describe complete neutralization and have also been observed with non-MPER antibodies [13], [39]. Antibody PG9 binds to one gp120 subunit on HIV-1 Env and occasionally partially neutralizes virus due to heterogeneity in glycan that forms part of its epitope [34]. However, PG9 has not been shown to occupy spikes that remain infectious, and 10E8 has no reported dependency on glycan. We did find reference to partial neutralization involving antibodies to respiratory syncytial virus surface glycoprotein [69], [70], however a basis for the effect was not proposed or further investigated.

10E8 has a reported weak affinity for membranes [29]. We also found evidence for weak binding of 10E8 to bald viral particles and cells that may warrant further investigation (unpublished results). However, autoreactivity has no clear correlation with neutralization potency [15], [25], [28], [71]. Importantly, our results show that quaternary structure and stability of HIV-1 Env also affects neutralization as well as antibody occupancy at the MPER.

For vaccine design, partial occupancy of Envs by MPER antibodies or B cell receptors (BCRs) elicited early in a primary response could alter the structure and immunogenicity of Env. Trimeric immunogens could be identified that promote or discourage specific quaternary features of the native MPER. Neutralizing antibodies that saturate all three MPERs of the HIV-1 spike in a clash free manner may be the most potent and therefore most desirable to elicit. Approaches to enhance immunogenicity of the MPER on native spikes are also desired, including prime-boost strategies using MPER specific immunogens equipped with compatible T cell epitopes [8], [9], [11], [12], [47].

Vaccination, immunotherapy and immunoprophylaxis are becoming increasingly attractive approaches to combat HIV/AIDS [1]–[3], [72], so 10E8 clearly warrants further investigation considering its extreme potency and breadth of neutralization. Our results raise, however, a potential caveat for monotherapy using 10E8 (or single epitope vaccines based on the 10E8 epitope) due to the potential for partial escape mutants to be functionally stabilized by 10E8 or 10E8-like antibodies. However, we also show that 10E8 partial-resistant mutants are hypersensitive to certain gp41 antibodies and fusion inhibitors in 10E8-bound form (**Figures 2** **and** **5**). Targeting multiple sites of vulnerability on gp41 and other conserved regions of Env will best capitalize on this heightened sensitivity and limit the possibility for neutralization escape.

In conclusion, our work shows that in order to gain a full picture of neutralization at the base of the trimeric spike, that consideration be given not only to the interaction antibody makes with a single MPER but also to the stability and recognition properties of adjacent, unoccupied MPERs and subunits of trimeric Env both pre- and post- receptor engagement.

## Materials and Methods

### Plasmids

HIV-1 backbone plasmids pSG3ΔEnv and pNL4-3.Luc.R^-^.E^-^ were obtained through the NIH AIDS Research and Reagent Program (ARRRP), contributed by J. Kappes and X. Wu and by N. Landau, respectively. Env complementation plasmid pSVIIIexE7pA^−^_YU2_ was kindly provided by J. Sodroski (Harvard) and the envelope genes JR2 [47] and SF162 [73] were cloned in pSVIIIexE7pA^−^ using the *KpnI* and *XhoI* sites as described previously [47]. Molecular clones of JR-FL, JR2 and SF162 were produced by subcloning into plasmid pLAI.2 as described previously [9]. Env plasmids COT6 and its MPER Ala mutants and TM20.13 were kindly provided by E. Gray and L. Morris (National Institute of Communicable Diseases, Johannesburg). Env plasmids M27390 PL 1706 and M20490 BMR 211 were kindly provided by G. Aldrovandi (Children's Hospital of Los Angeles) [49]. Quikchange mutagenesis was performed on JR-FL, JR2, SF162, COT6 and M27390 PL 1706 according to the manufacturer's protocol (Agilent).

### Antibodies and inhibitors

HIV-1 antibodies were obtained from the following sources (target epitope and subunit in parentheses): 10E8 (MPER, gp41) IgG heavy and light chain DNA expression vectors were kindly provided by M. Connors (VRC, NIH) and IgG was produced in house, Fab 10E8 was prepared using Endoproteinase Lys-C (Promega) digestion according to the manufacturer's protocol. IgGs 2F5 and 4E10 (MPER, gp41) were purchased from Polymun (Vienna). IgGs Z13e1 (MPER, gp41), 8K8 (NHR, gp41), and DN9 (NHR, gp41) [40] as well as 5-Helix (CHR, gp41) [74] were produced in house. IgGs PG9 (V2, gp120) [56], b12 (CD4 binding site, or CD4bs, gp120) [75] and b6 (CD4bs, gp120) [76] were generously provided by D. Burton (Scripps). PGT121 (N332 supersite, gp120) [77] was a gift from P. Poignard (Scripps). IgGs VRC01 (CD4bs, gp120) [78], and F425 B4e8 (V3 crown, gp120) [79], were obtained through the ARRRP, contributed by J. Mascola, and by M. Posner and L. Cavacini, respectively. IgG 17b (CD4bs, gp120) [80] was kindly provided by J. Robinson (Tulane). IgGs 2G12 (glycan, gp120) [81] and 447-52D (V3 crown, gp120) [82] were purchased from Polymun (Vienna). Soluble CD4 was purchased from Progenics (Tarrytown), and C34 peptide [83] was obtained through the ARRRP.

### Cell lines

HEK-293 cells were from the ATCC, and 293 GnTI^-^ cells were a gift from H.G. Khorana (MIT) [84]. TZM-bl cells (CD4^+^CXCR4^+^CCR5^+^), TZM-bl FcγRI cells [53], U87 cells (CD4^+^CCR5^+^), and HOS cells (CD4^+^CCR5^+^) were obtained through the ARRRP, contributed by J. Kappes and X. Wu, by D. Montefiori and G. Perez, by H. Deng and D. Littman, and by N. Landau, respectively. TZM-bl cell lines were maintained in DMEM supplemented with 10% FBS, 2 mM L-glutamine, 100 U of penicillin/ml, and 100 µ g/ml of streptomycin. U87.CCR5 cells lines were maintained in DMEM supplemented with 15% FBS, 2 mM L-glutamine, 100 U/ml of penicillin, and 100 µg/ml of streptomycin, 1 µg/ml puromycin, and 1 µg/ml G418. HOS cells were maintained in the same medium as TZM-bl but with the addition of 1 µg/ml puromycin.

### Virus production

Pseudotyped viruses were produced by transfection of HEK-293 cells. DNA comprising Env plasmid and pSG3ΔEnv or pNL4-3.Luc.R^-^.E^-^ at a mass ratio of 1 3.5 was mixed with transfection reagent polyethylene imine (PEI 25K, Sigma-Aldrich). Kifunensine (Cayman Chemical Co.) was added to HEK-293 cells 30 min prior to transfection [6]. Virus was alternatively produced in 293 GnTI- cells as described previously [85]. Virus containing supernatant was harvested 72 hours post transfection and 0.2 µm filtered to remove cellular debris. Viral supernatants were aliquoted and stored at −80°C.

### Neutralization assays

Single cycle viral entry neutralization assays were performed using TZM-bl cells, unless otherwise indicated. TZM-bl FcγRI cells, U87.CCR5 cells, and HOS cells were also used, as indicated. Cells (10^5^ per well) were seeded in 96 well plates 24 hours prior to assay. The virus and inhibitor mixture was incubated at 37°C, and then added to TZM-bl cells. Infectivity was measured 48 h post infection using a luciferase assay system (Promega) and a Synergy HT Microplate Reader (Bio-Tek). Data was processed using Prism 5.0 software (Graphpad). Washout neutralization assays were performed as described previously [35]. Time course neutralization assays were performed by allowing the virus and inhibitor mixture to incubate for 1, 8 or 20 hour(s) at 37°C. Maturation neutralization assays were performed using virus that had been pre-incubated at 37°C for 20 hours prior to usage. Neutralization assays in the presence of 10E8 were performed by adding to the virus and inhibitor mixture a constant concentration of 10E8 IgG, typically 10 µg/ml for MPER mutants and 0.1 µg/ml and 0.01 µg/ml for wild type JR-FL and SF162, respectively. The mixture of virus, 10E8 and inhibitor were incubated at 37°C for 1 hour and then added to TZM-bl cells. Modified assays were processed just as the infectivity assay described above.

To test the effect of 10E8 aggregation on neutralization activity, 10E8 IgG was deliberately aggregated through concentration with Amicon Ultra centrifugal filters (Millipore) in PBS. Aggregate was pelleted by centrifugation at 22,000×*g* for 5 min and the soluble fraction of 10E8 in the supernatant was saved. The pellet was washed 4 times in PBS by vortexing and vigorous pipetting throughout which time the aggregate remained visible and insoluble. The amount of 10E8 in the pellet was estimated by subtracting the amount recovered in the soluble fraction. Neutralization assays using 10E8 visibly aggregated in suspension were performed as described above.

### HIV-1 temperature gradient (T_90_) and half-life (t_1/2_) infectivity decay assays

Temperature gradient infectivity assays were determined using a gradient PCR block (Mastercycler, Eppendorf) as previously described [4]. Briefly, virus samples were incubated over a thermal gradient range from 37°C to 56°C for 1 hour in parallel using a 96 well PCR plate. Thermally treated virus samples were cooled to room temperature and added to TZM-bl cells. Luciferase activity was determined 48 hours post infection as described above. The temperature at which 10% of infectivity remained (T_90_) was determined using Prism 5.0 software (Graphpad, La Jolla). In half-life infectivity decay experiments, virus and antibody were co-incubated at 37°C for various time intervals and infectivity of virus was determined using TZM-bl indicator cells. Data was plotted using a non-linear, one phase exponential decay equation (plateau constraint  = 0) and t_1/2_ was determined using Prism 5.0 software.

### Blue native (BN) PAGE, gel mobility shift assay and western blot

Virions were produced by transfection using molecular clone plasmid pLAI, pelleted in an Optima ultracentrifuge (Beckman; 60,000×*g* at 4°C) and resuspended 100-fold concentrated in PBS. For gel mobility shift assays, virions were pre-incubated with antibodies for 30 min before preparation for BN-PAGE. In some cases virus was pelleted in a microcentrifuge for 45 min at 4°C and the buffer was exchanged to remove unbound antibody prior to detergent treatment. For heat gradient BN-PAGE, virions were exposed to a temperature gradient for 1 hour, as detailed above, prior to detergent solubilization. BN-PAGE was performed as previously described [4]. Briefly, samples were treated with 1% DDM for 20 min on ice. Samples were then electrophoresed on 3-12% NativePAGE Bis-Tris gels (Invitrogen) according to the manufacturer's instructions. Proteins in the gel were then transferred to a PVDF membrane; membranes were blocked in 5% non-fat dry milk and blotted overnight at 4°C using a cocktail of antibodies to gp120 (2 µg/ml each of b12, 2G12 and 447-52D) and to gp41 (1 µg/ml each of 2F5, 4E10 and Z13e1) combined. Membranes were washed, probed for 30 min at room temperature with a HRP conjugated goat anti-human Fc antibody (Jackson), and peroxidase activity was assayed using Super Signal West Pico Chemiluminescence (Pierce). Relevant exceptions to this protocol are noted in figure legends.

In order to quantify antibody stoichiometry, the distance between the midpoints of Fab-shifted vs untreated bands on BN-PAGE blots was measured using ImageJ software (NIH), and divided by the distance shifted by Fabs b12 and PG9 that are assumed to bind three and one Fab(s) per trimer, respectively. To estimate antibody affinity for the detergent-solubilized Env trimer, BN-PAGE blots were again analyzed using ImageJ software and the percentage of trimer that remained unshifted at each concentration was calculated by comparing the band intensity to that of samples with no antibody added.

## Supporting Information

Figure S1 **10E8 IgG partially neutralizes JR2 mutants W672A and F673L with a maximum plateau that is consistent between replicate experiments #1 and #2 despite presence or absence of a downward slope at very high concentrations of 10E8 and despite a tendency of 10E8 to aggregate.** (**A**) In experiment #1 the neutralization plateau remains stable whereas in experiment #2 the neutralization curve shows a plateau that is followed by a downward slope at high concentrations of 10E8. Each datum point in both experiments is an average of a duplicate with error bars shown, although error within experiment was extremely small. The experimental artifact or element responsible for the difference in curve shape between replicate experiments is currently undetermined. (**B**) 10E8 IgG was deliberately aggregated by concentration (see Materials and Methods) and as a visible aggregate in suspension is shown to produce similar partial neutralization activity as the soluble aggregate-free fraction of 10E8.
(TIFF)

Figure S2 **Effect of conservative or non-conservative substitutions at positions 672, 673 and 683 in the MPER on neutralization of HIV-1 by 10E8.** Selected conservative or non-conservative mutations to (**A**) F673, (**B**) W672, and (**C**) K683 were introduced into JR2 and the corresponding viruses tested for neutralization sensitivity to 10E8.
(TIFF)

Figure S3 **Partial neutralization of HIV-1 F673L by 10E8 is not restricted to TZM-bl cells.** Neutralization sensitivity of HIV-1 JR2 mutant F673L to MPER antibodies on (**A**) U87.CD4.CCR5 and (**B**) HOS.CD4.CCR5 as target cells. (**C**) Neutralization sensitivity of HIV-1 JR2 and mutant F673L with 10E8 IgG on TZM-bl FcγRI reporter cells. (**D**) Partial neutralization plateau percentages of 10E8 IgG on various target cells.
(TIFF)

Figure S4 **Glycosylation state of MPER mutant Env influences extent of maximum neutralization by 10E8.** (**A**) The four conserved N-glycosylation sites (NGS) in gp41 were individually mutated on a JR2 F673L Env background and corresponding viruses were tested in neutralization assays against 10E8 (left), 4E10 (middle) and 2F5 (right). (**B**) JR2 F673L virions, engineered with an E168K mutation to generate the PG9 epitope, were produced in the presence or absence of the glycosidase inhibitor kifunensine (Kif), which prevents the formation of complex glycans so glycosylation remains high mannose (*i.e.* Man_9_ residues). Viruses were tested against 10E8 (left), 2F5 (middle), and the Kif-sensitive antibody, PG9 (right) [56]. Mutation E168K had no effect on 10E8 neutralization (data not shown). (**C**) Neutralization of JR2 (right panels) and cognate F673L mutant (left panels) by 2F5, 10E8 and sCD4 using virus produced in either 293S (GnTI^-/-^) cells, a cell line that is unable to generate complex glycans so glycosylation comprises Man_5_ up to all Man_9_ residues (top panels), or 293T cells (bottom panels).
(TIFF)

Figure S5 **Kifunensine (Kif) treatment moderately impairs Env processing and function.** Replication competent JR2 wild-type (wt) and F673A virions were produced by transfection of 293T cells in the presence or absence of 25 µM Kif. (**A**) Relative infectivity of virions was analyzed by infection of TZM-bl target cells for 48 hours. (**B**) Apparent incorporation of gp120 was analyzed using a lectin-capture ELISA detected using gp120 antibodies (b12 and F425-B4e8). Results were normalized for p24 content as determined by p24 ELISA. (**C**) Cleavage efficiency of Env was determined using SDS-PAGE followed by Western blot with a cocktail of gp120 antibodies. Similar results were observed using gp41 antibodies.
(TIFF)

Figure S6 **Env trimers have greater thermal resistance in the presence of 10E8 but grow fainter on BN-PAGE Western blots without accumulation of decay products.** HIV-1 JR-FL wild type virions were heated at 37°C or 57°C for 1 hour in the presence or absence of 100 µg/ml Fab 10E8. Env was subsequently detergent-solubilized and analyzed using BN-PAGE. Western blots were stained using antibody cocktails specific for the gp41 MPER (4E10, 2F5 and Z13e1; left panel), gp41 cluster I and II epitopes (7B2, F240 and 98-6; middle panel), or gp120 (2G12, b12, 447-52D and b6; right panel). T = gp120/gp41 trimer.
(TIFF)

Figure S7 **10E8 maximum neutralization plateaus with HIV-1 mutant virus incubated at physiological conditions for an extended time period.** SF162 and JR2 F673 mutants were either used fresh or incubated at 37°C for 20 hours prior to the addition of 10E8. Incubated virus and antibody mixture was incubated for 1 hour at 37°C and then added to TZM-bl cells.
(TIFF)

Figure S8 **Neutralization of HIV-1 JR2 wild type and F673L mutant with and without ligand washout.** Wild type (left panel) and mutant F673L (right panel) virus were treated with inhibitors (**A**) 10E8, (**B**) C34, and (**C**) b12, and incubated 1 hour at 37°C. Virus was pelleted and washed to remove unbound inhibitor before adding to TZM-bl cells.
(TIFF)

Figure S9 **Cartoon models portraying possible mechanism of effects of 10E8 on HIV-1 Env trimer structure-function.** (**A**) The unliganded Env spike of SF162 F673 mutant in the absence or presence of 10E8 showing exposure of receptor binding site. *Left panel*: Wild type SF162 (not shown) and cognate F673L virus are fully sensitive to soluble CD4, b6 and 17b in the absence of 10E8, indicating spontaneous exposure of CD4bs (purple circle) and elements of the inner domain of gp120 (dark shading). *Right panel*: Binding by 10E8 (green) stabilizes conformations of the SF162 F673 spike in which inner domains of gp120 are less exposed (inward arrows) causing interference with the ability of b6 and 17b to neutralize the virus and reduced sensitivity to sCD4. (**B**) Prefusion intermediate of gp41 showing stabilizing effect of 10E8 on exposure of the heptad repeats of receptor-activated gp41. *Left panel*: The NHR coiled coil (yellow cylinders), CHR regions (blue strands), and MPER (red strands) of the metastable (blurred lines) pre-fusion intermediate of gp41, are sensitive to DN9/8K8/C34 (pink), 5-Helix (not shown), and MPER antibodies (*e.g.* 2F5 and Z13e1; not shown), respectively. *Right panel*: Binding by 10E8 (rightmost green) stabilizes conformations of the pre-fusion intermediate NHR and CHR regions of gp41 that are favorable for ligand binding, but less favorable for binding by other MPER antibodies (leftmost green). (**C**) Presence of 10E8 stabilizes function of MPER mutant Envs of SF162 and JR2. *Left panel*: Env spikes containing MPER mutations that destabilize unliganded Env (see Fig. 8; blurred lines). *Right panel*: Presence of Fab 10E8 (green) increases thermostability of the MPER mutant spikes in their receptor-naïve state. (**D**) Occupancy of wild type JR-FL and MPER mutant Env trimers by 10E8. *Left panel*: Fab 10E8 (green) readily occupies all three gp41 subunits (yellow) of wild type, unliganded Env, which also destabilizes the functional trimer over time. *Right panel*: Fab 10E8 (green) binds with less apparent affinity (not depicted) and with lower subunit occupancy (up to two Fabs per spike) for spikes containing the F673A mutation, which stabilizes the functional trimer. Partial subunit occupancy by 10E8 also limits exposure of the CD4 binding site and the 10E8-unbound MPER (depicted by an X), as in Fig. 2 and 4. We depict the 10E8-bound trimer in an asymmetric arrangement in which gp41 (yellow) and gp120 (blue) subunits have shifted (arrows); however, no structures of 10E8 bound spikes have been determined so this arrangement is merely for illustration and more subtle structural changes can also be envisioned.
(TIFF)

Table S1 HIV-1 isolates and variants used in this study with cognate amino acid sequences in the membrane proximal external region (MPER).
(DOCX)

Table S2 Effect of presence of 4E10 on the sensitivity of F673L mutants to MPER and CD4bs antibodies and inhibitors.
(DOCX)
