# Supplementary figures and images for: Antibody to gp41 MPER Alters Functional Properties of HIV-1 Env without Complete Neutralization
Source: PLoS Pathog. 2014 Jul 24;10(7):e1004271. doi: 10.1371/journal.ppat.1004271 (PMC4110039; doi:10.1371/journal.ppat.1004271)

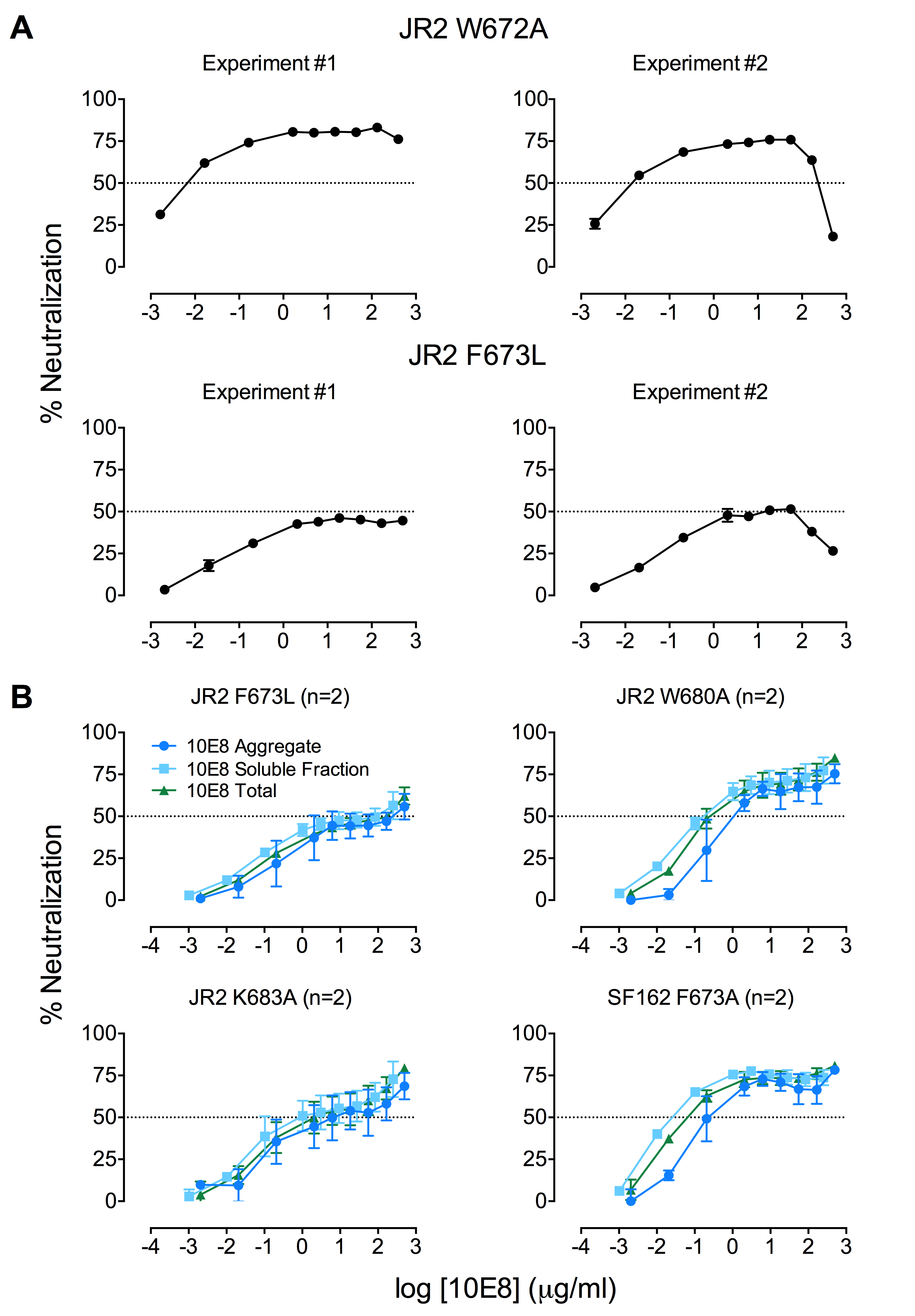

Supplement: Figure S1 — 10E8 IgG partially neutralizes JR2 mutants W672A and F673L with a maximum plateau that is consistent between replicate experiments #1 and #2 despite presence or absence of a downward slope at very high concentrations of 10E8 and despite a tendency of 10E8 to aggregate. (A) In experiment #1 the neutralization plateau remains stable whereas in experiment #2 the neutralization curve shows a plateau that is followed by a downward slope at high concentrations of 10E8. Each datum point in both experiments is an average of a duplicate with error bars shown, although error within experiment was extremely small. The experimental artifact or element responsible for the difference in curve shape between replicate experiments is currently undetermined. (B) 10E8 IgG was deliberately aggregated by concentration (see Materials and Methods) and as a visible aggregate in suspension is shown to produce similar partial neutralization activity as the soluble aggregate-free fraction of 10E8. (TIFF) [file ppat.1004271.s001.tiff]

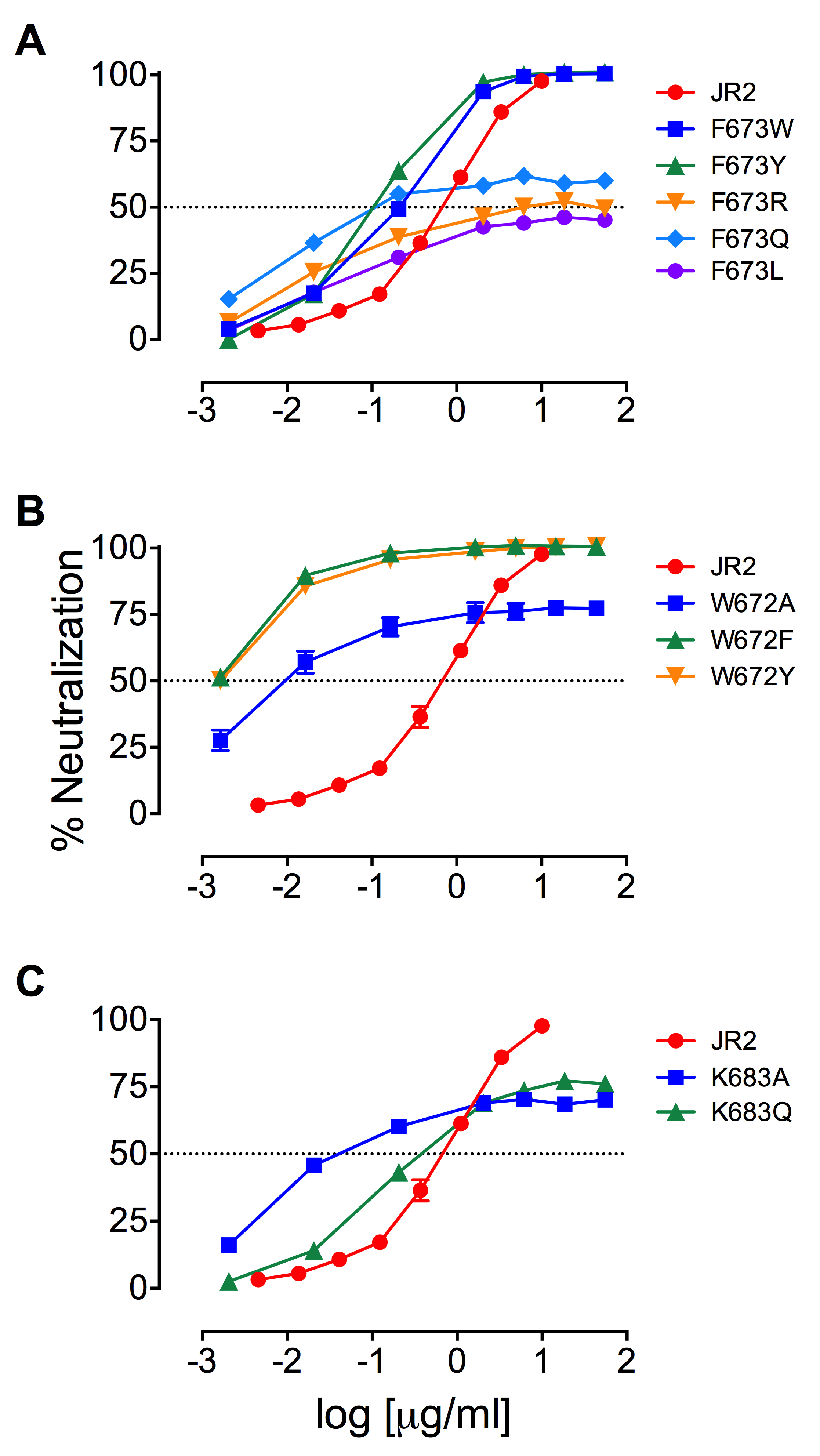

Supplement: Figure S2 — Effect of conservative or non-conservative substitutions at positions 672, 673 and 683 in the MPER on neutralization of HIV-1 by 10E8. Selected conservative or non-conservative mutations to (A) F673, (B) W672, and (C) K683 were introduced into JR2 and the corresponding viruses tested for neutralization sensitivity to 10E8. (TIFF) [file ppat.1004271.s002.tiff]

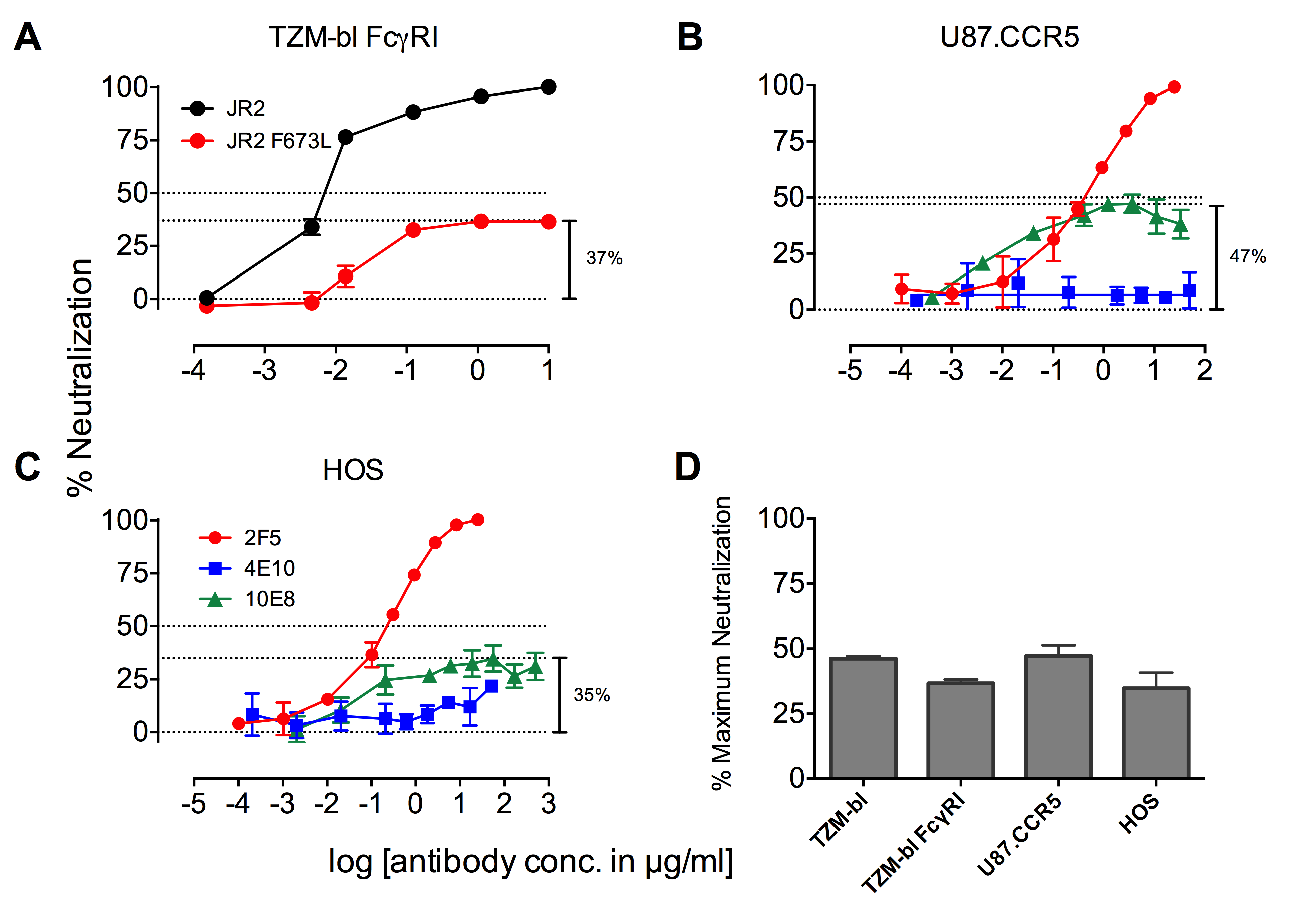

Supplement: Figure S3 — Partial neutralization of HIV-1 F673L by 10E8 is not restricted to TZM-bl cells. Neutralization sensitivity of HIV-1 JR2 mutant F673L to MPER antibodies on (A) U87.CD4.CCR5 and (B) HOS.CD4.CCR5 as target cells. (C) Neutralization sensitivity of HIV-1 JR2 and mutant F673L with 10E8 IgG on TZM-bl FcγRI reporter cells. (D) Partial neutralization plateau percentages of 10E8 IgG on various target cells. (TIFF) [file ppat.1004271.s003.tiff]

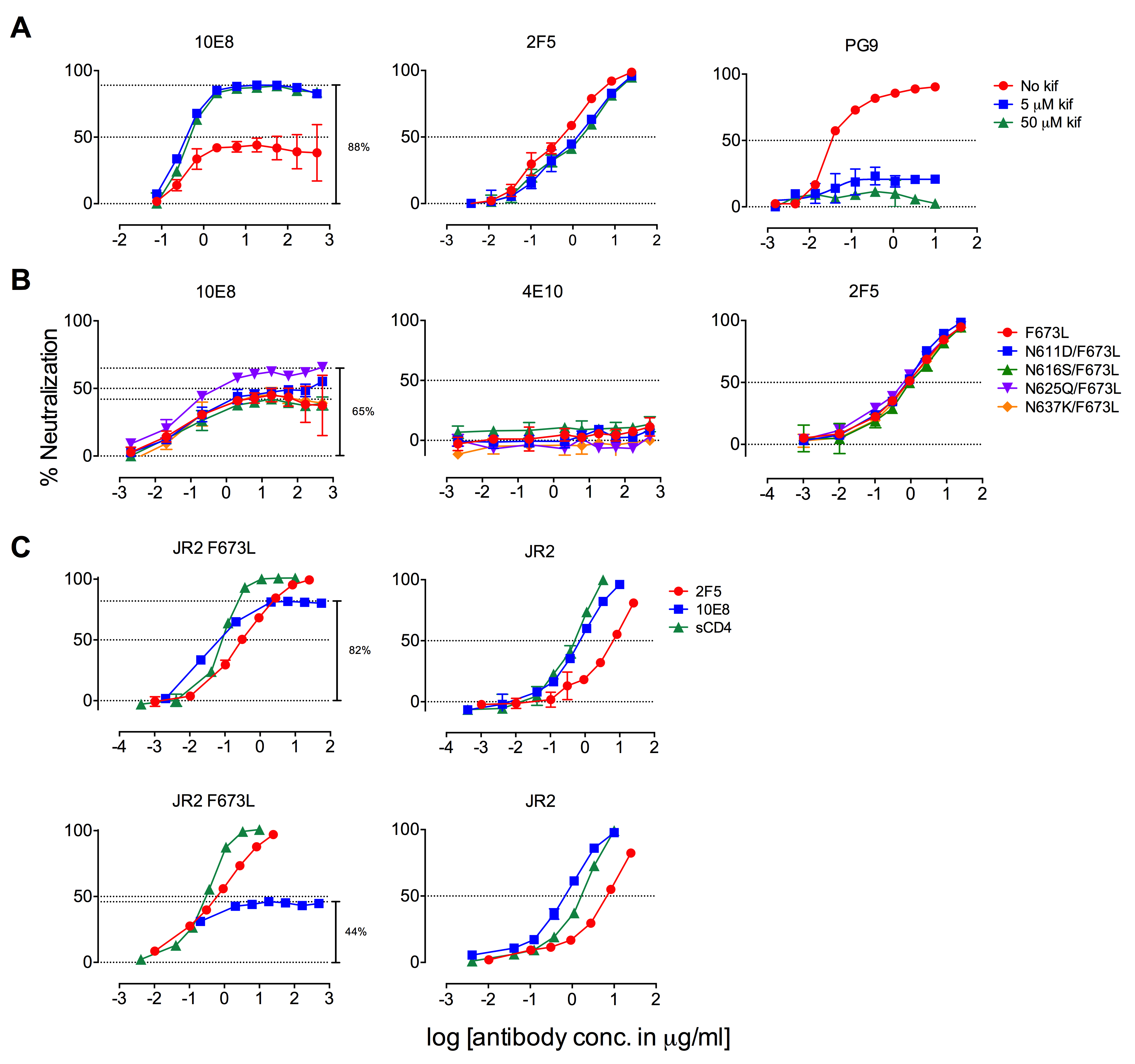

Supplement: Figure S4 — Glycosylation state of MPER mutant Env influences extent of maximum neutralization by 10E8. (A) The four conserved N-glycosylation sites (NGS) in gp41 were individually mutated on a JR2 F673L Env background and corresponding viruses were tested in neutralization assays against 10E8 (left), 4E10 (middle) and 2F5 (right). (B) JR2 F673L virions, engineered with an E168K mutation to generate the PG9 epitope, were produced in the presence or absence of the glycosidase inhibitor kifunensine (Kif), which prevents the formation of complex glycans so glycosylation remains high mannose (i.e. Man9 residues). Viruses were tested against 10E8 (left), 2F5 (middle), and the Kif-sensitive antibody, PG9 (right) [56]. Mutation E168K had no effect on 10E8 neutralization (data not shown). (C) Neutralization of JR2 (right panels) and cognate F673L mutant (left panels) by 2F5, 10E8 and sCD4 using virus produced in either 293S (GnTI-/-) cells, a cell line that is unable to generate complex glycans so glycosylation comprises Man5 up to all Man9 residues (top panels), or 293T cells (bottom panels). (TIFF) [file ppat.1004271.s004.tiff]

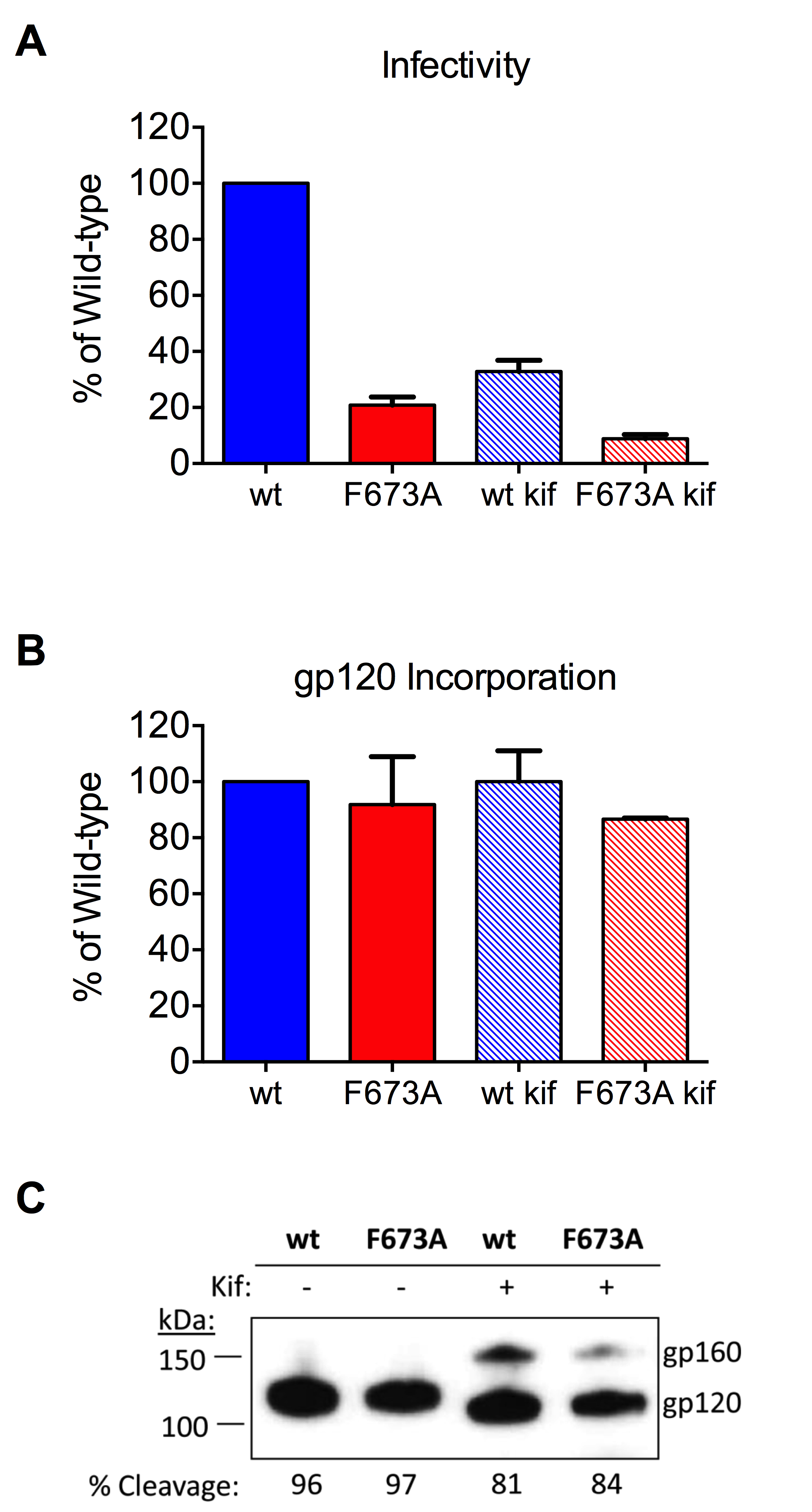

Supplement: Figure S5 — Kifunensine (Kif) treatment moderately impairs Env processing and function. Replication competent JR2 wild-type (wt) and F673A virions were produced by transfection of 293T cells in the presence or absence of 25 µM Kif. (A) Relative infectivity of virions was analyzed by infection of TZM-bl target cells for 48 hours. (B) Apparent incorporation of gp120 was analyzed using a lectin-capture ELISA detected using gp120 antibodies (b12 and F425-B4e8). Results were normalized for p24 content as determined by p24 ELISA. (C) Cleavage efficiency of Env was determined using SDS-PAGE followed by Western blot with a cocktail of gp120 antibodies. Similar results were observed using gp41 antibodies. (TIFF) [file ppat.1004271.s005.tiff]

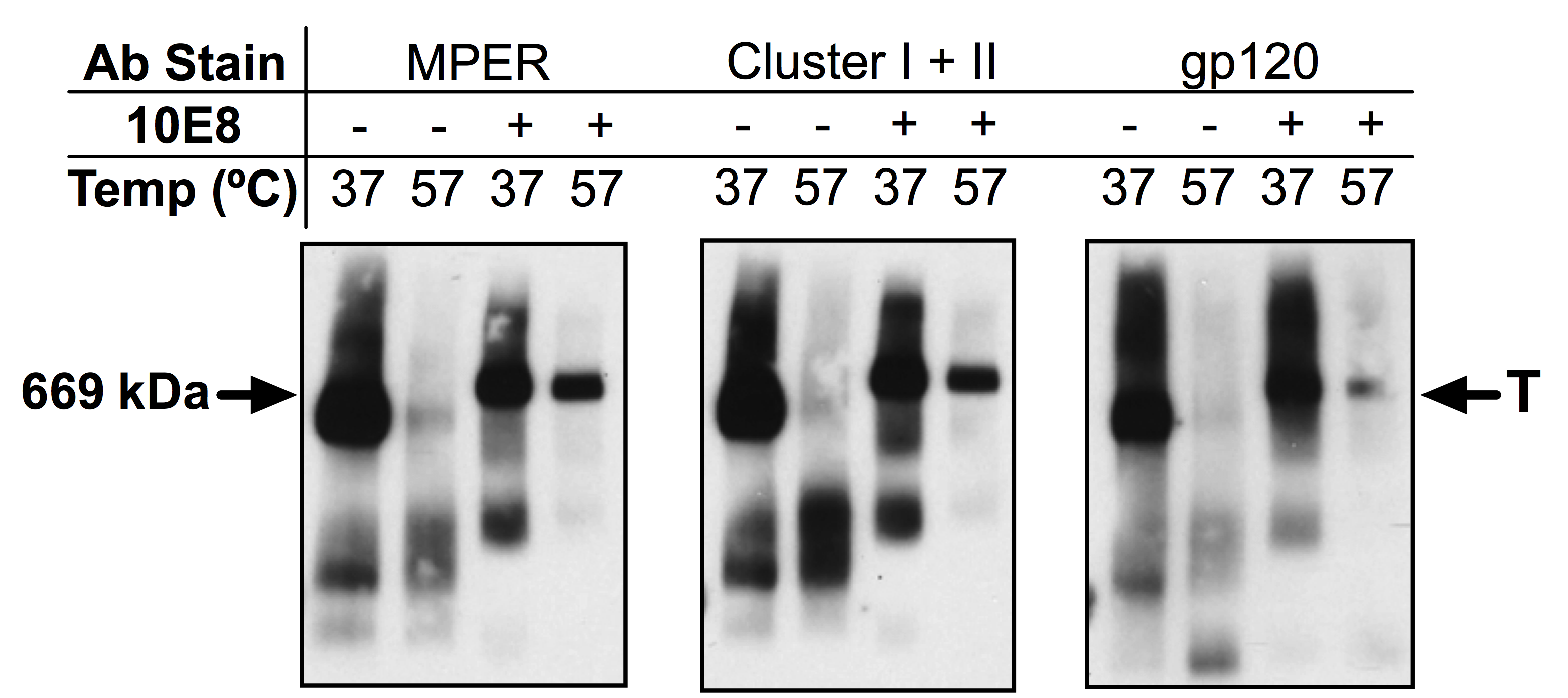

Supplement: Figure S6 — Env trimers have greater thermal resistance in the presence of 10E8 but grow fainter on BN-PAGE Western blots without accumulation of decay products. HIV-1 JR-FL wild type virions were heated at 37°C or 57°C for 1 hour in the presence or absence of 100 µg/ml Fab 10E8. Env was subsequently detergent-solubilized and analyzed using BN-PAGE. Western blots were stained using antibody cocktails specific for the gp41 MPER (4E10, 2F5 and Z13e1; left panel), gp41 cluster I and II epitopes (7B2, F240 and 98-6; middle panel), or gp120 (2G12, b12, 447-52D and b6; right panel). T = gp120/gp41 trimer. (TIFF) [file ppat.1004271.s006.tiff]

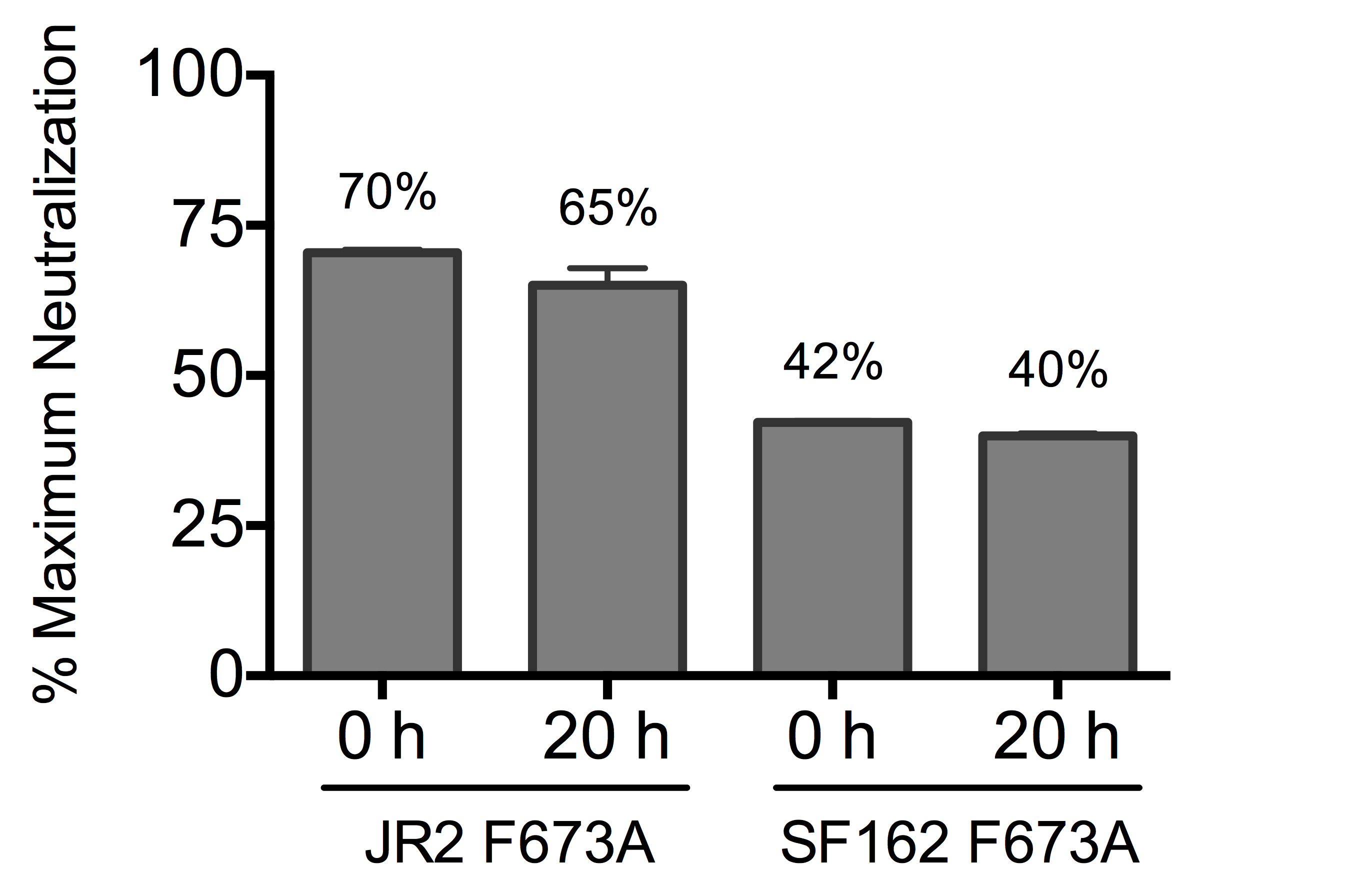

Supplement: Figure S7 — 10E8 maximum neutralization plateaus with HIV-1 mutant virus incubated at physiological conditions for an extended time period. SF162 and JR2 F673 mutants were either used fresh or incubated at 37°C for 20 hours prior to the addition of 10E8. Incubated virus and antibody mixture was incubated for 1 hour at 37°C and then added to TZM-bl cells. (TIFF) [file ppat.1004271.s007.tiff]

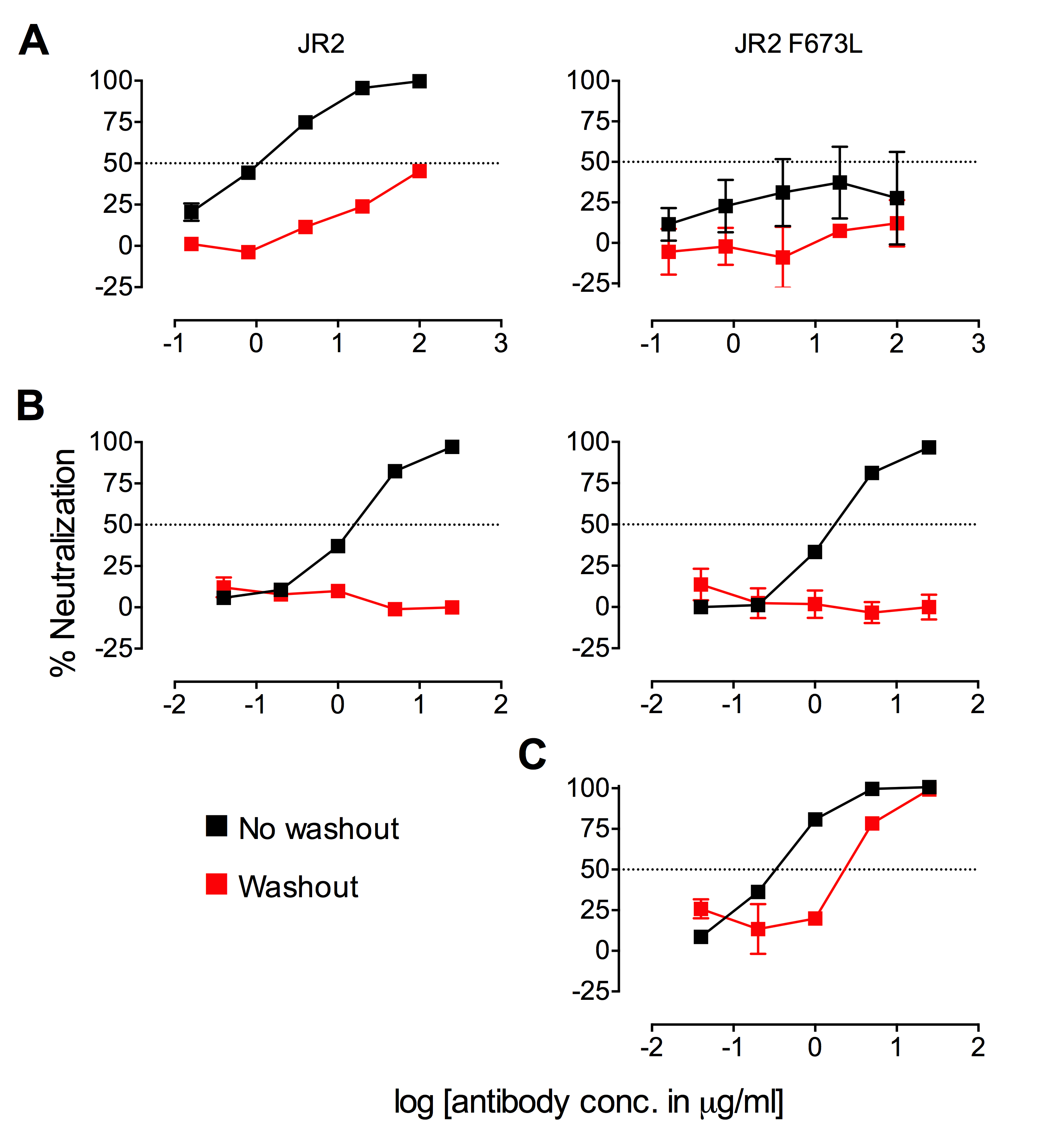

Supplement: Figure S8 — Neutralization of HIV-1 JR2 wild type and F673L mutant with and without ligand washout. Wild type (left panel) and mutant F673L (right panel) virus were treated with inhibitors (A) 10E8, (B) C34, and (C) b12, and incubated 1 hour at 37°C. Virus was pelleted and washed to remove unbound inhibitor before adding to TZM-bl cells. (TIFF) [file ppat.1004271.s008.tiff]

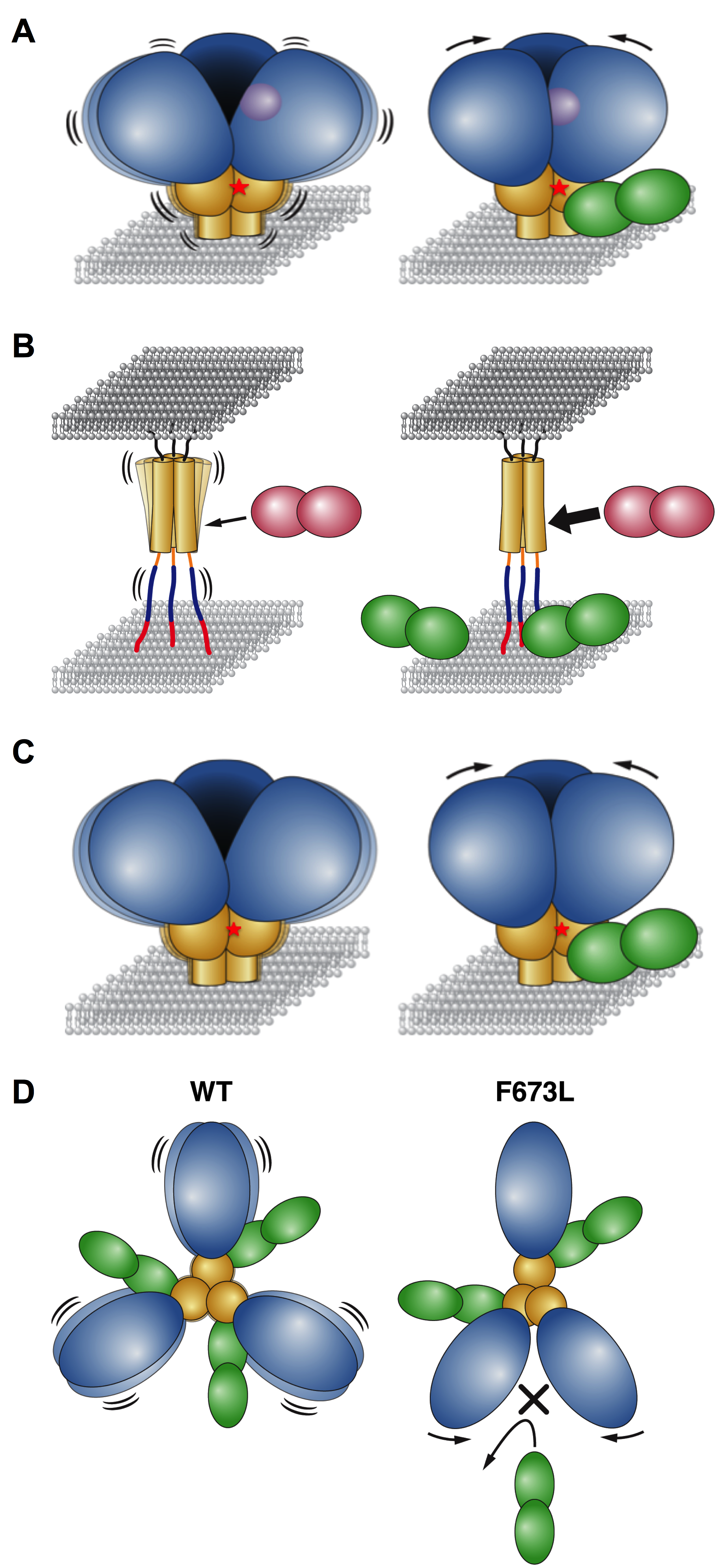

Supplement: Figure S9 — Cartoon models portraying possible mechanism of effects of 10E8 on HIV-1 Env trimer structure-function. (A) The unliganded Env spike of SF162 F673 mutant in the absence or presence of 10E8 showing exposure of receptor binding site. Left panel: Wild type SF162 (not shown) and cognate F673L virus are fully sensitive to soluble CD4, b6 and 17b in the absence of 10E8, indicating spontaneous exposure of CD4bs (purple circle) and elements of the inner domain of gp120 (dark shading). Right panel: Binding by 10E8 (green) stabilizes conformations of the SF162 F673 spike in which inner domains of gp120 are less exposed (inward arrows) causing interference with the ability of b6 and 17b to neutralize the virus and reduced sensitivity to sCD4. (B) Prefusion intermediate of gp41 showing stabilizing effect of 10E8 on exposure of the heptad repeats of receptor-activated gp41. Left panel: The NHR coiled coil (yellow cylinders), CHR regions (blue strands), and MPER (red strands) of the metastable (blurred lines) pre-fusion intermediate of gp41, are sensitive to DN9/8K8/C34 (pink), 5-Helix (not shown), and MPER antibodies (e.g. 2F5 and Z13e1; not shown), respectively. Right panel: Binding by 10E8 (rightmost green) stabilizes conformations of the pre-fusion intermediate NHR and CHR regions of gp41 that are favorable for ligand binding, but less favorable for binding by other MPER antibodies (leftmost green). (C) Presence of 10E8 stabilizes function of MPER mutant Envs of SF162 and JR2. Left panel: Env spikes containing MPER mutations that destabilize unliganded Env (see Fig. 8; blurred lines). Right panel: Presence of Fab 10E8 (green) increases thermostability of the MPER mutant spikes in their receptor-naïve state. (D) Occupancy of wild type JR-FL and MPER mutant Env trimers by 10E8. Left panel: Fab 10E8 (green) readily occupies all three gp41 subunits (yellow) of wild type, unliganded Env, which also destabilizes the functional trimer over time. Right panel: Fab 10E8 (green) binds w [file ppat.1004271.s009.tiff]
