# Supplementary material for: Antibody to gp41 MPER Alters Functional Properties of HIV-1 Env without Complete Neutralization
Source: PLoS Pathog. 2014 Jul 24;10(7):e1004271. doi: 10.1371/journal.ppat.1004271 (PMC4110039; doi:10.1371/journal.ppat.1004271)
Supplement: Table S1 — HIV-1 isolates and variants used in this study with cognate amino acid sequences in the membrane proximal external region (MPER). (DOCX) [file ppat.1004271.s010.docx]

**Table S1. HIV-1 isolates and variants used in this study with cognate amino acid sequences in the membrane proximal external region (MPER).**

| **HIV-1 Variant** | **MPER Sequence (aa 662-683)*** | | | **Reference** | |
| --- | --- | --- | --- | --- | --- |
| **JR2** | ELDKWANLWNWFDISNWLWYIK | | | Zwick et al., 2005 | |
| N671A | .........A............ | | | Zwick et al., 2005 | |
| N671T | .........T............ | | | Ingale et al., 2010 | |
| W672A | ..........A........... | | | Zwick et al., 2005 | |
| F673A | ...........A.......... | | | Zwick et al., 2005 | |
| F673L | ...........L.......... | | | This study | |
| W680A | ................A..... | | | Zwick et al., 2005 | |
| W680G | ................G..... | | | This study | |
| K683A | .....................A | | | This study | |
| K683Q | .....................Q | | | This study | |
|  |  |  |  |  |  |
| **JRFL** | ELDKWASLWNWFDITKWLWYIK | | | Zwick et al., 2005 | |
| F673A | ...........A.......... | | | This study | |
| F673L | ...........L.......... | | | This study | |
|  |  |  |  |  |  |
| **SF162** | ELDKWASLWNWFDISKWLWYIK | | | Cheng-Mayer et al., 1988 | |
| F673A | ...........A.......... | | | This study | |
| F673L | ...........L.......... | | | This study | |
| W680G | ..................G... | | | This study | |
|  |  |  |  |  |  |
| **M27390 PL 1706** | ALDSWKNLWNWFDISKWLWYIK | | | Nakamura et al., 2010 | |
| F673L | ...........L.......... | | | This study | |
|  |  |  |  |  |  |
| **M20490 BMR 211** | ALDSWKNLWNWLSISKWLWYIK | | | Nakamura et al., 2010 | |
|  |  |  |  |  |  |
| **TM20.13** | ALDKWNNLWSWLSISNWLWYIK | | | Gray et al., 2008 | |
|  |  |  |  |  |  |
| **COT6** | ALDSWKNLWSWFDITKWLWYIK | | | Gray et al., 2007 | |
| F673A | ...........A.......... | | | Gray et al., 2007 | |

*HxB2 numbering, aa, amino acids
