# Supplementary material for: Antibody to gp41 MPER Alters Functional Properties of HIV-1 Env without Complete Neutralization
Source: PLoS Pathog. 2014 Jul 24;10(7):e1004271. doi: 10.1371/journal.ppat.1004271 (PMC4110039; doi:10.1371/journal.ppat.1004271)
Supplement: Table S2 — Effect of presence of 4E10 on the sensitivity of F673L mutants to MPER and CD4bs antibodies and inhibitors. (DOCX) [file ppat.1004271.s011.docx]

**Table S2. Effect of presence of 4E10 on the sensitivity of F673L**

**mutants to MPER and CD4bs antibodies and inhibitors.**

| **Inhibitor** | **Site of inhibition** | **Fold change in IC_50_ due to 4E10*** | | | |
| --- | --- | --- | --- | --- | --- |
|  |  | **JR2 F673L** | | **SF162 F673L** | |
| **2F5** | gp41 MPER | 1.0 | | 1.2 | |
| **10E8** | gp41 MPER | *na*^1,2^ | | 1.0^2^ | |
| **Z13e1** | gp41 MPER | 0.8 | | 0.9 | |
|  |  |  |  |  |  |
| **sCD4** | gp120 CD4bs | *nd*^3^ | | 0.5 | |
| **b12** | gp120 CD4bs | *nd*^3^ | | 1.5 | |

*Fold change in IC_50_ = (IC_50_ without 4E10 / IC_50_ with 4E10). ^1^*na*, not applicable, as an IC_50_ was not reached at concentrations tested. ^2^Partial neutralization dose response curve observed. ^3^Not determined.
